# Identification of proliferative progenitors associated with prominent postnatal growth of the pons

**DOI:** 10.1038/ncomms11628

**Published:** 2016-05-18

**Authors:** Robert A. Lindquist, Cristina D. Guinto, Jose L. Rodas-Rodriguez, Luis C. Fuentealba, Matthew C. Tate, David H. Rowitch, Arturo Alvarez-Buylla

**Affiliations:** 1 Eli & Edythe Broad Center of Regeneration Medicine and Stem Cell Research, University of California—San Francisco, San Francisco, California 94143, USA; 2 Medical Scientist Training Program, University of California—San Francisco, San Francisco, California 94143, USA; 3 Neuroscience Graduate Program, University of California—San Francisco, San Francisco, California 94143, USA; 4 Department of Neurological Surgery, University of California—San Francisco, San Francisco, California 94143, USA; 5 Department of Pediatrics, University of California—San Francisco, San Francisco, California 94143, USA

## Abstract

The pons controls crucial sensorimotor and autonomic functions. In humans, it grows sixfold postnatally and is a site of paediatric gliomas; however, the mechanisms of pontine growth remain poorly understood. We show that the murine pons quadruples in volume postnatally; growth is fastest during postnatal days 0–4 (P0–P4), preceding most myelination. We identify three postnatal proliferative compartments: ventricular, midline and parenchymal. We find no evidence of postnatal neurogenesis in the pons, but each progenitor compartment produces new astroglia and oligodendroglia; the latter expand 10- to 18-fold postnatally, and are derived mostly from the parenchyma. Nearly all parenchymal progenitors at P4 are Sox2^+^Olig2^+^, but by P8 a Sox2^−^ subpopulation emerges, suggesting a lineage progression from Sox2^+^ ‘early' to Sox2^−^ ‘late' oligodendrocyte progenitor. Fate mapping reveals that >90% of adult oligodendrocytes derive from P2–P3 Sox2^+^ progenitors. These results demonstrate the importance of postnatal Sox2^+^Olig2^+^ progenitors in pontine growth and oligodendrogenesis.

## Abstract (web summary)

Postnatal growth of the pons is not well characterized. Here the authors show that growth of the murine pons is fastest during postnatal day 0–4, a period preceding myelination, and is primarily driven by an expansion of the oligodendrocyte population that derive from Sox2^+^Olig2^+^ progenitors.

## Graphical abstract

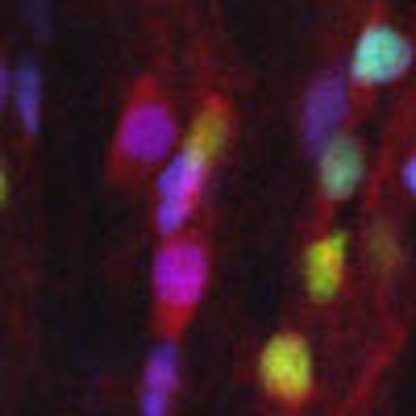

The mammalian brain undergoes significant postnatal growth. Postnatal growth could aid experience-dependent acquisition of brain function^1^, and may confer susceptibility to tumorigenesis^2^. The human pons is a site of exceptional postnatal growth^3^ and lethal paediatric gliomas^4^; however, knowledge of the cells responsible for pons growth remains limited. One study suggested a ‘second peak' of Nestin^+^ progenitor cells in ventral human pons at 7 years^5^, though a subsequent study did not find evidence to support that claim, and instead observed that such progenitors were restricted to infancy^3^. The nature of postnatal pontine progenitor cells has consequences for pontine gliomagenesis, and for the normal postnatal development and function of this crucial brain region.

The dorsal pons (tegmentum) contains autonomic nuclei controlling awakeness and arousal^6^, respiration^7^ and adrenergic tone^8^, as well as cranial nerves and nuclei mediating facial sensation and motion^9^. The ventral pons (basis pontis) contributes to motor function, in part as a ‘bridge' between cortex and cerebellum: neurons in the pontine grey nuclei receive synapses from cortex and project to cerebellum via the middle cerebellar peduncle^10^. Interestingly, among primates the size of the basis pontis scales directly with the size of the neocortex^11^. The basis pontis also contains the corticospinal tract, whose descending axons carry motor signals from cortex to spinal motor neurons. Many pontine circuits serve functions that are postnatally acquired or refined, but nearly all studies of pons development have focused on prenatal events, beginning with its embryonic derivation from various segments of the rhombencephalon. Pontine neurogenesis occurs prenatally^12^,^13^,^14^,^15^,^16^,^17^: autonomic nuclei and cranial nerves (V–VIII) derive mainly from rhombomeres 2 to 5 (refs 18, 19), though some tegmental neurons derive from rhombomere 1 (ref. 20); the neurons of the basis pontis are born in rhombomeres 6–8 and migrate anteriorly to form the pontine grey nuclei in the territory of rhombomeres 3–4 (refs 20, 21). Proliferation^22^ and gliogenesis^23^,^24^,^25^,^26^ have been documented in embryonic murine and avian pons, but the question of which cells are responsible for postnatal pontine growth remains unanswered.

Here we show that as in humans, the postnatal mouse basis pontis grows more than tegmentum, with growth comparable to rapidly growing forebrain structures; growth is fastest at birth and mostly occurs before P16. Postnatal progenitor cells in the pons are found (1) in the ventricular zone along the ventral walls of the fourth ventricle, (2) in the midline domain and (3) in the parenchyma. Progenitors proliferate in a single wave peaking at postnatal day 4. We observe that proliferative Olig2^+^ progenitors include an unexpected and abundant Sox2^+^ subpopulation, which is enriched in basis pontis. Remarkably, postnatal Sox2^+^ progenitor cells produce more than 90% of adult mouse pons oligodendrocytes, contributing to a 10- to 18-fold postnatal expansion of the oligodendroglial lineage.

## Results

### Postnatal pontine growth rates are fastest from P0 to P4

**Figure 1:**
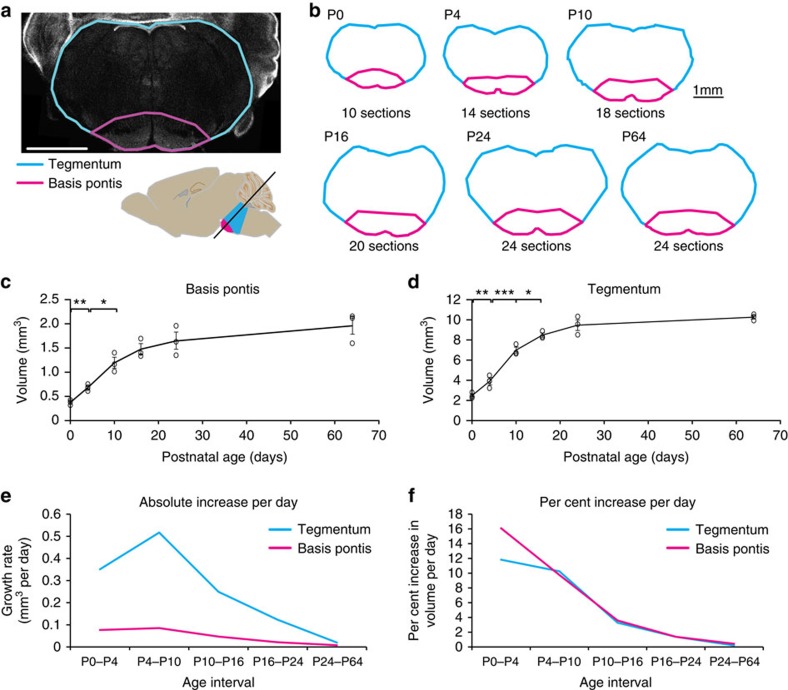
Postnatal growth of mouse pons. (**a**) DAPI stain of P0 mouse pons section containing tegmentum (cyan) and basis (magenta), sectioned at oblique angle normal to axis of spinal cord (inset). Basis pontis includes pontine nuclei and adjacent white matter tracts (transversus fasciculus pontis, longitudinalis fasciculus pontis, and middle cerebral peduncle ventral to pontine nuclei). Scale bar=1 mm. (**b**) Postnatal growth of pons in dorsal-ventral and medial-lateral axes is illustrated by section tracings as in (**a**); growth along rostral-caudal axis is indicated by the number of sections passing through pons. Scale bar=1 mm. (**c**,**d**) Volumes of pons regions were computed by summation of areas measured in every second section through pons, times distance between measured sections (100 μm). Graphs show individual replicates with mean±s.e.m., *n*≥3 mice per timepoint. Unpaired *t*-test revealed significant differences between the following consecutive timepoints: P0 and P4 (both regions), P4 and P10 (both regions), and P10 and P16 (tegmentum). *P<0.05, **P<0.01, ***P<0.001. (**e**) Absolute increase in volume per day computed as (mean volume at end of interval—mean volume at beginning of interval)÷(number of days in interval). (**f**) Per cent increase in volume per day computed as (100 × ((mean volume at end of interval÷mean volume at beginning of interval)^(1/number of days in interval)^−1)).

The human pons grows sixfold from birth to adulthood^3^. To measure the postnatal growth of the mouse pons, we prepared CD1 wild-type mouse tissue for sectioning in a plane containing basis pontis, tegmentum and fourth ventricle (Fig. 1a and Methods section). We computed volumes of basis pontis and tegmentum based on areas measured in every second section through the pons (Fig. 1b–d). The basis pontis grew fivefold postnatally, from 0.38 mm^3^ at birth to 1.96 mm^3^ in adulthood (Fig. 1c), while the pontine tegmentum grew fourfold, from 2.48 mm^3^ to 10.3 mm^3^ (Fig. 1d). This growth was comparable to the four- to fivefold postnatal increase in mouse hippocampal and neocortical volume measured by magnetic resonance imaging^27^ and histologic methods^28^, and greater than the two- to threefold postnatal increase in volume of anterior thalamic nuclei^28^. The absolute volumetric growth rate (mm^3^ per day) was greater in tegmentum than in basis pontis, due to the tegmentum's larger size, and in tegmentum the absolute rate of growth was greatest during P4–P10 (Fig. 1e). However, the daily per cent increase in volume was greatest during P0–P4, and was greater in basis pontis than tegmentum during that period, with basis pontis growing at an average rate of 16% daily (Fig. 1f). The rate of growth then declined gradually through weaning age. The size of the pons at P24 was not significantly different from that in adults aged P64 (*P*>0.2, unpaired *t*-test). These data indicate that the mouse pons grows rapidly during early postnatal life and proportionally more in basis pontis than tegmentum.

### Myelination does not account for pontine growth from P0 to P4

**Figure 2:**
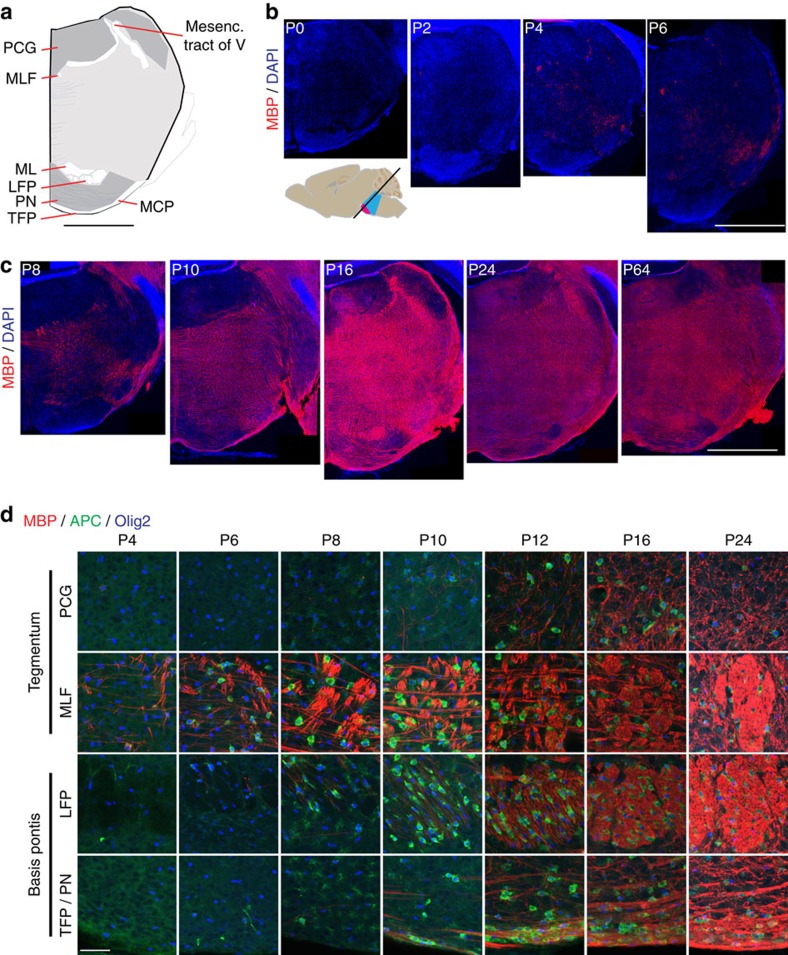
Progressive myelination in pons. (**a**) P16 pons section stained for myelin basic protein (MBP) was traced to highlight landmark tracts and grey/white regions. Lines indicate thick fibres within grey matter regions. (**b**,**c**) MBP-stained pons hemisections at indicated ages; red, MBP; blue, DAPI. Scale bar for (**a**–**c**)=1 mm. (**d**) High-resolution images of representative pons regions, including both grey and white matter within tegmentum and basis, illustrate differently timed regional onsets of myelination and progressive increase over postnatal development. Red, MBP; green, APC; blue, Olig2. Scale bar, 50 μm. LFP, longitudinal fasciculus pontis; MCP, middle cerebellar peduncle; ML, medial lemniscus; MLF, medial longitudinal fasciculus; PCG, pontine central grey; PN, pontine nuclei; TFP, transverse fasciculus pontis.

The rapid early postnatal growth of human basis pontis is attributable in part to myelination^3^. To determine whether myelination accounted for the rapid early growth of the postnatal mouse basis pontis, we performed immunohistochemistry for myelin basic protein (MBP). The distribution of MBP in pontine sections of increasing age is shown in Fig. 2a–c. MBP appeared in concert with adenomatous polyposis coli (APC), a marker of mature oligodendrocytes, first in selected white matter tracts of tegmentum, for example, medial longitudinal fasciculus and trigeminal nerve (V) by P2–P4, followed by white matter of basis pontis from P8 to P10 and grey matter of basis pontis and tegmentum by P8 to P12 (Fig. 2d). The extent of myelination and the apparent thickness of myelinated tracts increased throughout the pons between P10 and P24 (Fig. 2c,d). The progression of myelination evidenced by MBP staining is consistent with a magnetic resonance imaging study on the development of 12 selected white matter tracts in postnatal mouse brain (including three brainstem tracts)^27^. Interestingly, while our data show that the pons underwent its most rapid growth before P4, hardly any MBP fibres were observed in P4 basis pontis (Fig. 2b,d). This suggests that, in contrast to human, the mouse pons shows rapid early postnatal growth before extensive addition of myelin.

### Proliferation occurs in three germinal zones and peaks at P4

**Figure 3:**
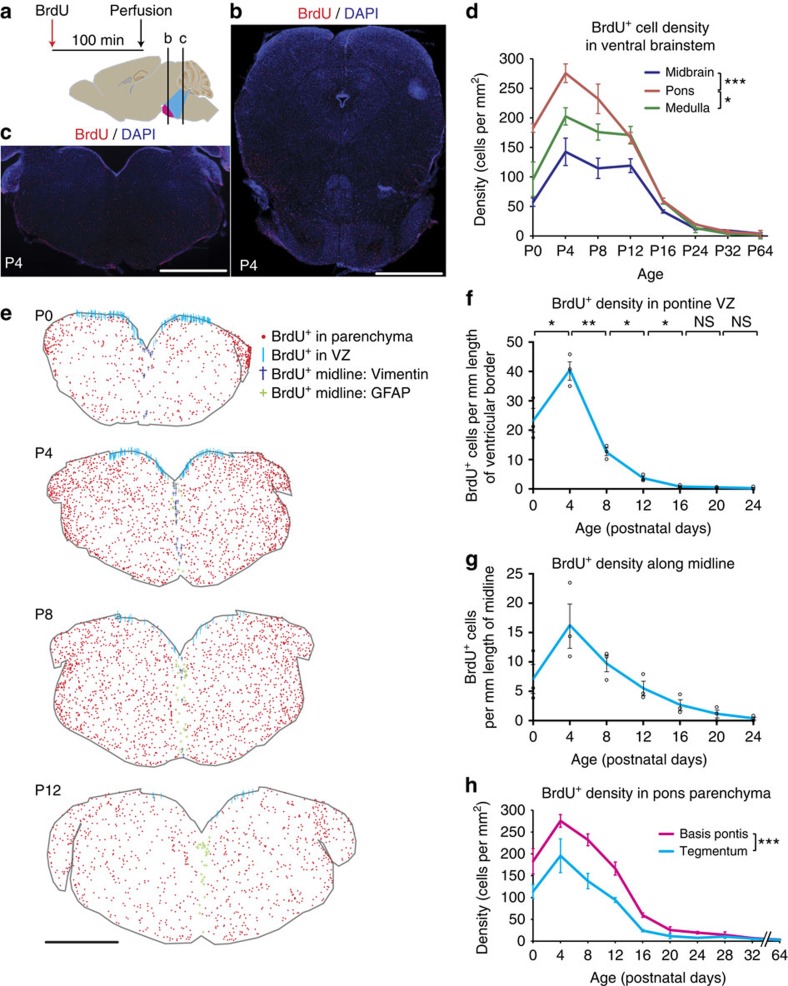
BrdU reveals three compartments of pontine proliferation. (**a**) CD1 wild-type mice were given a single dose of BrdU by intraperitoneal injection (50 mg kg^−1^) 100 min before perfusion, thereby labelling acutely proliferative cells. Standard coronal sections indicate levels of basis pontis (**b**) and pontine 4th VZ (**c**). (**b**,**c**) DAPI and BrdU costains at levels shown in (**a**) reveal proliferation throughout the mouse brainstem, but concentrated in basis pontis (**b**) and along 4th ventricle (**c**). Scale bar, 1 mm. (**d**) Density of BrdU^+^ cells is greater in basis pontis (ventral pons) than in ventral midbrain (*F*_1,32_=78.16, *P*<0.0006) or ventral medulla (*F*_1,32_=11.53, *P*=0.0108; regional comparisons by two-way ANOVA and Bonferroni *post hoc* adjustment; graph shows mean±s.e.m., *n*=3 mice per timepoint). See Supplementary Fig. 3 for dorsal brainstem regions. (**e**) Representative sections at level of (**c**) traced in StereoInvestigator software. Each mark represents one BrdU^+^ nucleus. Cyan marks represent BrdU^+^ cells in VZ, which was defined by the density of DAPI along the 4th ventricle; midline populations were defined by contact with vimentin (dark blue) or GFAP (green) fibres that contacted the midline glial structure; all other BrdU^+^ nuclei were considered parenchymal and labelled red. Scale bar, 1 mm. (**f**) Density of proliferative cells along pontine VZ; graph shows individual replicates with mean±s.e.m. (*n*=3 mice per timepoint); comparisons are by unpaired *t*-test. (**g**) Density of BrdU^+^ cells along midline; graph shows individual replicate values with mean±s.e.m. (*n*=3 mice per timepoint). (**h**) Density of BrdU^+^ cells is greater in basis pontis than pontine tegmentum (*F*_1,40_=39.06, *P*<0.0006; regional comparisons by two-way ANOVA and Bonferroni *post hoc* adjustment; graph shows mean±s.e.m., *n*=3 mice per timepoint). *P<0.05; **P<0.01, ***P<0.001. NS, not significant.

On the basis of the findings above, we hypothesized that early postnatal pontine growth could be due to proliferation of neuronal and/or glial precursor cells. To measure proliferation in postnatal development, we injected an age series of CD1 wild-type mice with a single dose of the thymidine analogue BrdU, 100 min before perfusion (Fig. 3a). Immunostaining revealed BrdU^+^ cells throughout the mouse brainstem (Fig. 3b,c); the density of BrdU^+^ cells was greater in basis pontis than in any other region of the brainstem (Fig. 3d,h; Supplementary Fig. 3f,g), and also greater in the pontine tegmentum relative to midbrain tegmentum (Supplementary Fig. 3h). We observed three main anatomical compartments of BrdU^+^ cells: (1) along the ventral surface of the fourth ventricle (ventricular zone, ‘VZ'); (2) along the midline; and (3) within parenchyma (Fig. 3e). In all three compartments, the density of BrdU^+^ cells was greatest at P4 (Fig. 3f–h), coinciding with the period of fastest pons growth (Fig. 1e,f). Notably, in the parenchymal compartment, BrdU^+^ cells were denser in the basis pontis than in the tegmentum (Fig. 3h). This greater proliferation in basis pontis coincides with its larger daily per cent increase in volume (Fig. 1f). Basis pontis proliferation peaked at 276±15 cells per mm^2^ at P4, and at P12 was still more than half its P4 peak, while tegmentum proliferation peaked at 196±39 cells per mm^2^ at P4, and declined more than 50% by P12 (Fig. 3h). Similarly, proliferation in VZ and midline peaked at P4 with 40.6±3.1 and 16.3±3.7 cells per mm, respectively, and declined more than 50% by P8 and P12, respectively (Fig. 3f,g). There was a significant increase in proliferation density from P0 to P4 in VZ (*P*=0.0302, unpaired *t*-test) and basis pontis (*P*=0.0072), while in tegmentum and midline the increased density of BrdU^+^ cells from P0 to P4 was not statistically significant (*P*=0.128 and *P*=0.121, respectively). Parenchymal proliferation declined by P20 to <10% of its P4 peak, and in adulthood (P64) amounted to 1.5% of its P4 peak (Fig. 3h). VZ proliferation declined sooner, reaching <10% of its P4 peak by P12 and 1.5% of its P4 peak by P20 (Fig. 3f). We observed no BrdU^+^ VZ cells at P28 or above. These findings show that the pons is the most proliferative postnatal brainstem region; its cell proliferation shows a single postnatal peak, at P4, coincident with the period of fastest growth. These data also show that proliferation declines at different rates in pontine VZ, parenchyma, and midline, and at different levels of the brainstem.

### Neurogenesis does not occur in the postnatal pons

The birthdating studies of Altman and Bayer^12^,^13^,^14^,^15^,^16^,^17^ indicate that neurons of the rat pons are primarily born before birth. However, it is not known if some pontine neurons are born postnatally in mouse. Given the dramatic postnatal growth (Fig. 1) and the presence of some migrating precerebellar neurons entering the pontine grey nuclei as late as P0 (ref. 29), it was possible that some pontine neurons could be born postnatally. We delivered BrdU in a single dose to wild-type mice aged P0 or P4. Analysis at P28 revealed no BrdU^+^NeuN^+^ cells in either basis pontis or tegmentum; since this BrdU dosage regimen might have been insufficient to identify slowly or rarely dividing cells, we administered BrdU or EdU every 12 h over a 4-day course spanning ages P1–P4, P5–P8 (Supplementary Fig. 4a), P9–P12 and so on through P29–P32, followed by 28-day chase periods. (The use of EdU in fate mapping may have reduced survival of EdU-labelled cells on a second cell cycle post labelling^30^,^31^, but would not have affected cells undergoing a single cell cycle.) Many cells retained BrdU and/or EdU after 28-day chase, including some glial fibrillary acidic protein (GFAP)^+^ astrocytes and APC^+^ oligodendrocytes (Supplementary Fig. 4b,c); yet we observed no EdU^+^NeuN^+^ or BrdU^+^NeuN^+^ cells in the pons (Supplementary Fig. 4d). Finally, we performed co-staining of the neuroblast markers doublecortin (DCX) and PSA-NCAM with BrdU in mice aged P0, P4, P8… through P32, each perfused 100 min after a single dose of BrdU, and found no colocalization of BrdU with either DCX or PSA-NCAM (Supplementary Fig. 4e,f). Altogether, these results indicate that pontine neurons are not born postnatally in mice, and thus neurogenesis does not account for postnatal pontine growth.

### Postnatal expansion of the oligodendroglial lineage

**Figure 4:**
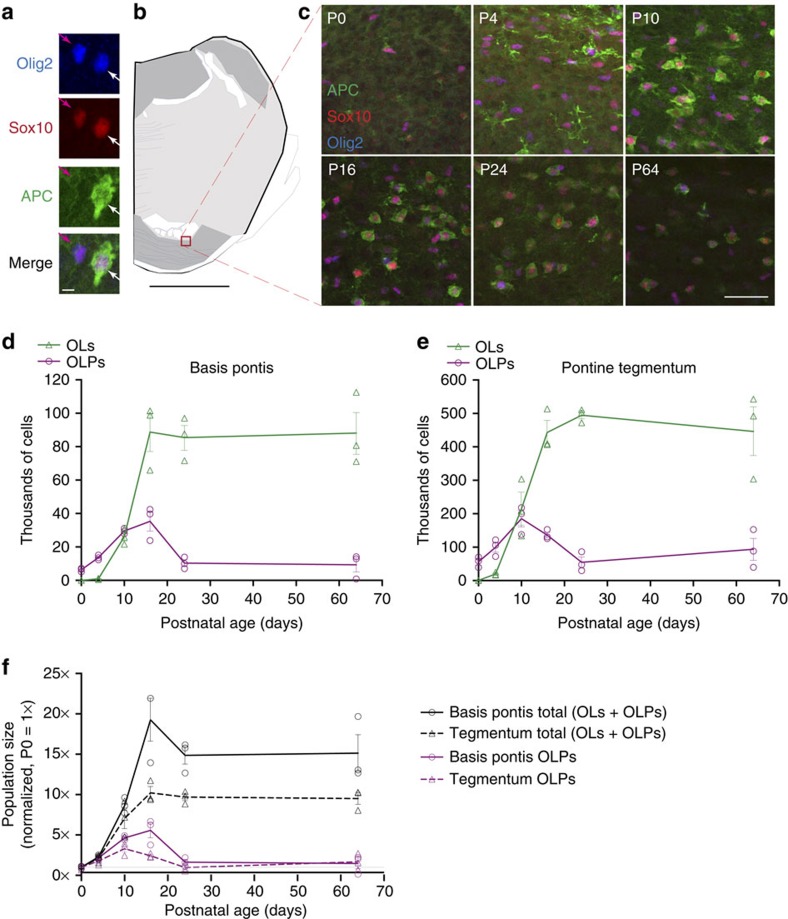
Expansion and maturation of OL lineage in postnatal pons. CD1 wild-type mouse pons sections were stained for Sox10, Olig2 and APC. (**a**) Representative OLP (Olig2^+^Sox10^+^APC^−^, magenta arrow) and OL (Olig2^+^Sox10^+^APC^+^, white arrow) from P10 mouse basis pontis. Scale bar, 5 μm. (**b**) Schematic of basis pontis region shown at several ages in (**c**). Scale bar, 1 mm. (**c**) Age series from basis pontis shows postnatal changes in density and maturation state of OLPs and OLs. Note sequential upregulation, condensation, and diminution of APC over time. Scale bar, 50 μm. (**d**,**e**) Stereologic counts of pons oligodendroglia in basis pontis (**d**) and tegmentum (**e**) over postnatal development, measured by Optical Fractionator probe in StereoInvestigator software. Cells were identified as OLs if they expressed APC and either or both of Sox10/Olig2; cells were counted as OLPs if they expressed both Sox10 and Olig2 but not APC. (**f**) Counts of OLPs (purple) and total oligodendroglia (OLs+OLPs, black) were normalized to P0 values, revealing fold expansion of the oligodendroglial population since P0 (1 ×). All graphs show individual replicates with mean±s.e.m. of *n*=3 mice per timepoint.

Because the pons lacks postnatal neurogenesis and undergoes delayed but extensive myelination, we hypothesized that pons growth may coincide with an increase in oligodendroglia. To count the oligodendroglial population in the developing postnatal pons, we performed stereologic analysis of wild-type tissue sections co-stained for Sox10, Olig2 and APC (Fig. 4a–f). Oligodendrocyte precursor cells (OLPs) were defined as Sox10^+^Olig2^+^APC^−^; oligodendrocytes (OLs) were defined as APC^+^ cells coexpressing Sox10, Olig2 or both (Fig. 4a). At birth, the basis pontis contained 6,357±610 OLPs, increasing to 13,688±790 OLPs by P4 and 29,527±922 by P10; OLPs peaked at P16 at 35,369±5,808, and declined by P24 to 10,372±2,004 (Fig. 4d). The number of mature OLs in basis pontis increased markedly from 958±212 at P4 to 26,119±2,666 at P10 and 88,750±11,417 at P16, after which the number of mature OLs in basis pontis remained constant to adulthood (Fig. 4d). The tegmentum underwent similar phases: the number of OLs in tegmentum was 19,990±2,612 at P4, increasing to 214,996±49,232 by P10 and 443,480±35,041 by P16 (Fig. 4e). The density of OLPs and morphology of APC staining changed markedly through postnatal development (Fig. 4c), reflecting the progressive increase and maturation of oligodendroglia through three phases: OLP expansion without differentiation (P0–P4), OLP expansion and differentiation to OLs (P4–P16), and OLP decline (P16–P24). Between P0 and P16, the oligo-lineage population increased 18-fold in basis pontis and 10-fold in tegmentum (Fig. 4f). The stereologic count of oligodendroglia, therefore, revealed a marked postnatal increase in oligodendroglia, and raised the question of which progenitor domains give rise to these new cells.

### Postnatal VZ and midline domains add few cells to parenchyma

**Figure 5:**
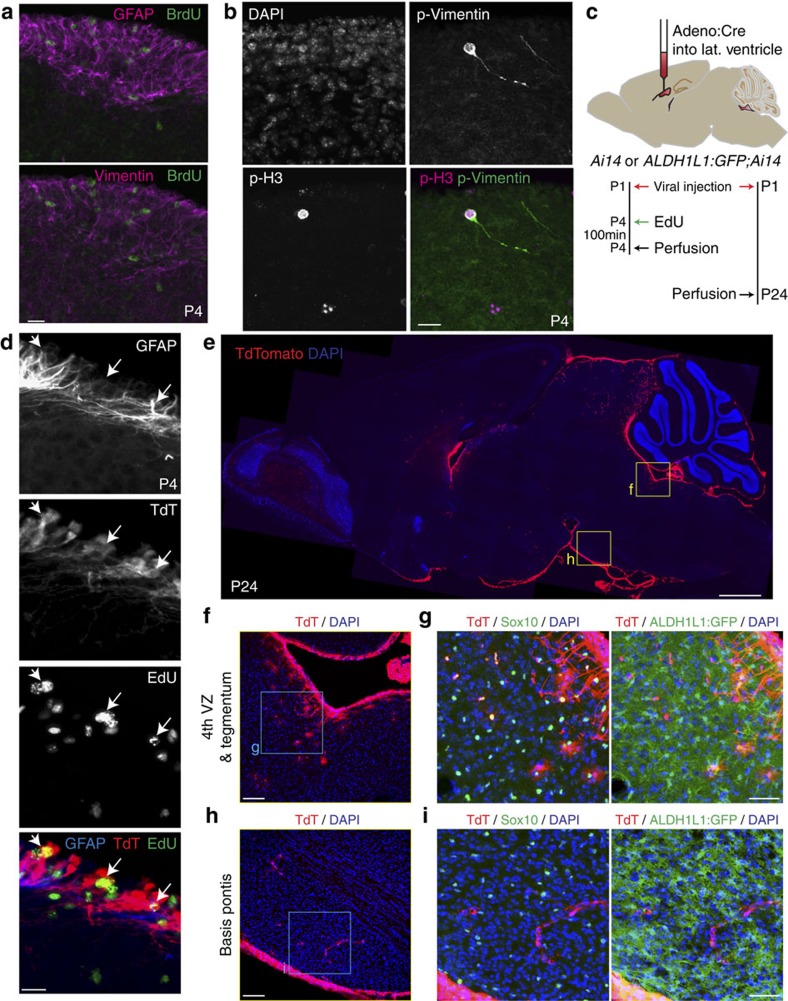
Properties and progeny of postnatal fourth VZ progenitor cells. (**a**) Wild-type mice received BrdU in a single dose 100 min before perfusion. BrdU^+^ nuclei in postnatal 4th VZ are surrounded by fibres of GFAP and vimentin. Scale bar, 20 μm. (**b**) Co-staining for M-phase marker phospho-histone-H3 (pH3) and phospho-ser55-Vimentin (p-Vimentin) reveals radial glial-like morphology of 4th VZ progenitors. Scale bar, 20 μm. (**c**–**i**) Viral fate mapping of postnatal 4th VZ progenitors. *Ai14* or *Ai14;ALDH1L1:GFP* pups received Adeno-Cre viral injection in lateral ventricle at age P1, and were perfused at P4 (100 min after a single dose of EdU) or at P24. (**d**) Co-staining of P4 tissue sections for EdU, GFAP, and TdTomato reveals proliferative TdT^+^ nuclei in the VZ of the 4th ventricle. Scale bar, 20 μm. (**e**) Cre-recombined cells are visible in olfactory bulb, confirming successful targeting of forebrain V-SVZ progenitors; along the surface of the entire ventricular system, including pontine 4th ventricle; and along the ventral surface of the brain, suggesting viral outflow to meningeal space via cisterna magna. Scale bar, 1 mm. (**f**) Enlarged view of 4th VZ and pontine tegmentum region indicated in (**e**), revealing parenchymal TdTomato (TdT)^+^ cells. Scale bar, 100 μm. (**g**) Further enlargment of pontine tegmentum as indicated in (**f**), showing co-immunostaining for TdTomato (Red) and lineage-specific markers (Green: left, Sox10; right, ALDH1L1:GFP). Scale bar, 50 μm. (**h**) Enlarged view of basis pontis region indicated in (**e**), revealing parenchymal TdT^+^ cells. Scale bar, 100 μm. (**i**) Further enlargment of basis pontis as indicated in **h**, showing immunostaining for TdT and lack of colocalization with either Sox10 (left) or ALDH1L1:GFP (right). Many TdT^+^ cells are found along structures morphologically consistent with blood vessels. Scale bar, 50 μm.

**Figure 6:**
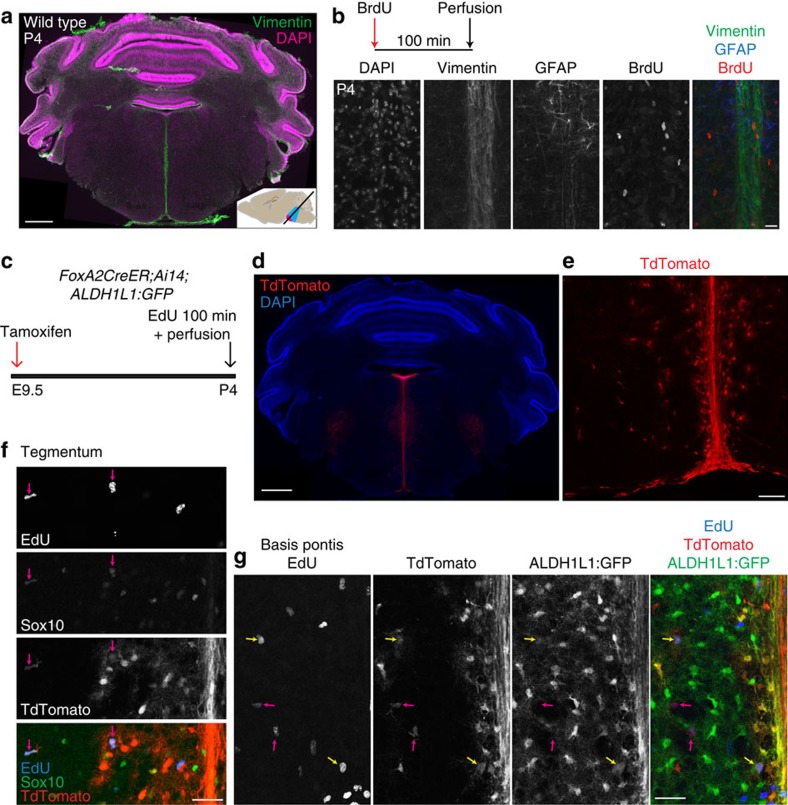
Proliferative progenitors in postnatal pons deriving from midline domain. (**a**) Wild-type mouse pons sectioned at oblique angle and stained for vimentin (green) and DAPI (magenta). Note the intense vimentin staining along the midline. Scale bar=500 μm. (**b**) Pons tissue from a mouse perfused 100 min after a single dose of BrdU (50 mg kg^−1^) was co-stained for Vimentin, GFAP and BrdU, revealing proliferating cells among fibres of the midline. Scale bar, 20 μm. (**c**) Schematic of fate mapping of midline domain. (**d**) TdTomato labelling by *FoxA2CreER;Ai14* in caudal pons, extending from fourth ventricle along midline, and including nearby cells. The midline and nearby cells were clearly labelled, along with other pontine structures known to derive from FoxA2^+^ progenitors (ref. 67): the motor nuclei of cranial nerves V, VI and VII, and raphe nuclei. Scale bar, 500 μm. (**e**) TdTomato expression in fibres along midline and in nearby parenchyma of basis pontis. Scale bar, 100 μm. (**f**) Co-labelling of TdTomato, EdU, and Sox10 reveals P4 proliferative oligodendroglia in tegmentum labelled by *FoxA2CreER* recombination at E9.5. Magenta arrows indicate Sox10^+^EdU^+^TdT^+^ cells. Scale bar, 50 μm. (**g**) Co-labelling of TdTomato, EdU, and ALDH1L1:GFP reveals that some P4 proliferative astrocytes are labelled by *FoxA2CreER* recombination at E9.5. Yellow arrows indicate ALDH1L1:GFP^+^EdU^+^TdT^+^ cells; magenta arrows indicate GFP^−^EdU^+^TdT^+^ cells. Scale bar, 50 μm. (**d**–**g**) In a section of bilateral width 4 mm, we observed no TdT^+^ EdU^+^ cells further than 500 μm from the midline; the only TdT^+^ cell bodies beyond 1 mm from midline were trigeminal motor neurons. These results show that while the midline domain produces postnatally proliferative astroglia and oligodendroglia, these progeny are regionally restricted.

Co-immunostaining for BrdU and intermediate filaments revealed proliferative progenitors associated with GFAP^+^ and Vimentin^+^ fibres in the postnatal VZ and midline domains (Figs 5a and 6a,b). Both VZ and midline also contained Nestin^+^ cells at P4 (Supplementary Fig. 5), and a subpopulation of VZ progenitors at M-phase showed phospho-Ser55-Vimentin staining in cells with radial glial-like morphology (Fig. 5b). The presence of proliferative cells and progenitor markers led us to ask whether these domains contribute new cells, and particularly oligodendroglia, to the growing pons parenchyma. We performed viral fate mapping of VZ progenitors by delivering adenoviral Cre into the lateral ventricles of P1 *Ai14* or *Ai14;ALDH1L1:GFP* mice^32^,^33^, thereby labelling VZ cells throughout the entire ventricular system (including fourth ventricle) with the TdTomato red fluorescent protein (Fig. 5c–e), and labelling also a small number of tegmental ALDH1L1:GFP^+^ astrocytes and Sox10^+^ oligodendroglia (Fig. 5e–i). We performed a similar labelling of mitotic, Nestin^+^ VZ progenitors by delivering RCAS:GFP retrovirus to the lateral ventricles of P2 mice carrying the *Nestin-tva* allele^34^, and again observed a small number of tegmental oligodendroglia close to the fourth ventricle (Supplementary Fig. 6). To label the midline domain, we used the *FoxA2CreER* driver^35^ crossed to the *Ai14* reporter, and observed TdTomato expression in midline glial fibres as well as a small subpopulation of nearby astroglia and oligodendroglia (Fig. 6c–g). Altogether, these experiments showed that while both VZ and midline domains produced both oligodendroglia and astroglia, their parenchymal progeny were few in number and remained close to VZ or midline.

### OLPs are the most proliferative population in postnatal pons

**Figure 7:**
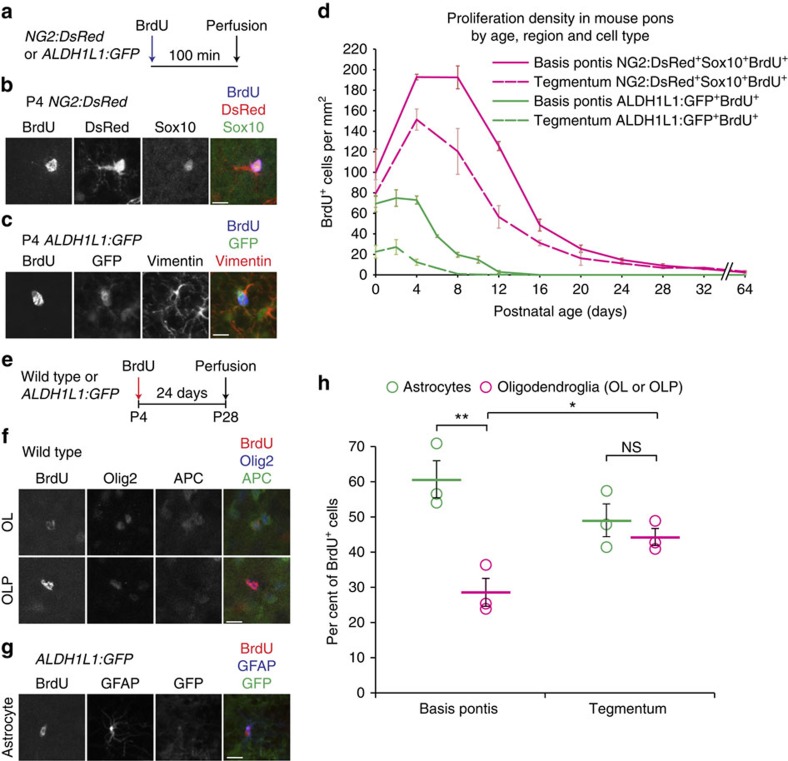
Glial lineages of parenchymal proliferative progenitors. (**a**–**d**) Mice carrying lineage-specific reporter transgenes received a single dose of BrdU (50 mg kg^−1^) 100 min before perfusion. (**b**) Representative proliferative OLP (Sox10^+^DsRed^+^BrdU^+^) in P4 *NG2:DsRed* mouse basis pontis. Scale bar, 10 μm. (**c**) Representative proliferative parenchymal astrocyte (BrdU^+^GFP^+^) in P4 *ALDH1L1:GFP* mouse basis pontis, with vimentin^+^ processes suggestive of colocalization. Scale bar, 10 μm. (**d**) Densities of proliferative OLPs from *NG2:DsRed* mice (red), and proliferative parenchymal astrocytes from *ALDH1L1:GFP* mice (green), at ages through childhood and at adulthood; mean±s.e.m., *n*=3 mice per strain per timepoint. Three-factor ANOVA confirmed significant differences in proliferation based on cell type (*F*_1,56_=491.97, *P*<0.0001), region (*F*_1,56_=70.18, *P*<0.0001), and age (*F*_9,56_=97.97, *P*<0.0001). (**e**–**h**) Wild-type or *ALDH1L1:GFP* mice were given a single dose of BrdU (50 mg kg^−1^) at P4 and perfused at P28. (**f**) BrdU^+^ cells from basis pontis of mice prepared as in **e**. Top, representative mature oligodendrocyte (Olig2^+^APC^+^BrdU^+^); bottom, representative OLP (Olig2^+^APC^−^BrdU^+^). Scale bar, 10 μm. (**g**) A representative GFAP^+^ALDH1L1:GFP^+^BrdU^+^ astrocyte from basis pontis of an *ALDH1L1:GFP* mouse that was given BrdU at P4 and perfused at P28. Scale bar, 10 μm. (**h**) Quantitation of BrdU^+^ astrocytes and oligodendroglia detected in pons regions after pulse at P4 and chase to P28; graph shows individual replicates with mean±s.e.m., *n*=3 mice per strain. Unpaired *t*-test shows BrdU^+^ astrocytes outnumber BrdU^+^ oligodendroglia in basis pontis (*P*=0.0099) and are in equal quantity to BrdU^+^ oligodendroglia in tegmentum (*P*=0.4320), while oligodendroglia are a lower fraction of BrdU^+^ cells in P28 basis pontis than tegmentum (See text for interpretations, *P*=0.0369). *P<0.05; **P<0.01; NS *P*>0.05. NS, not significant.

**Figure 8:**
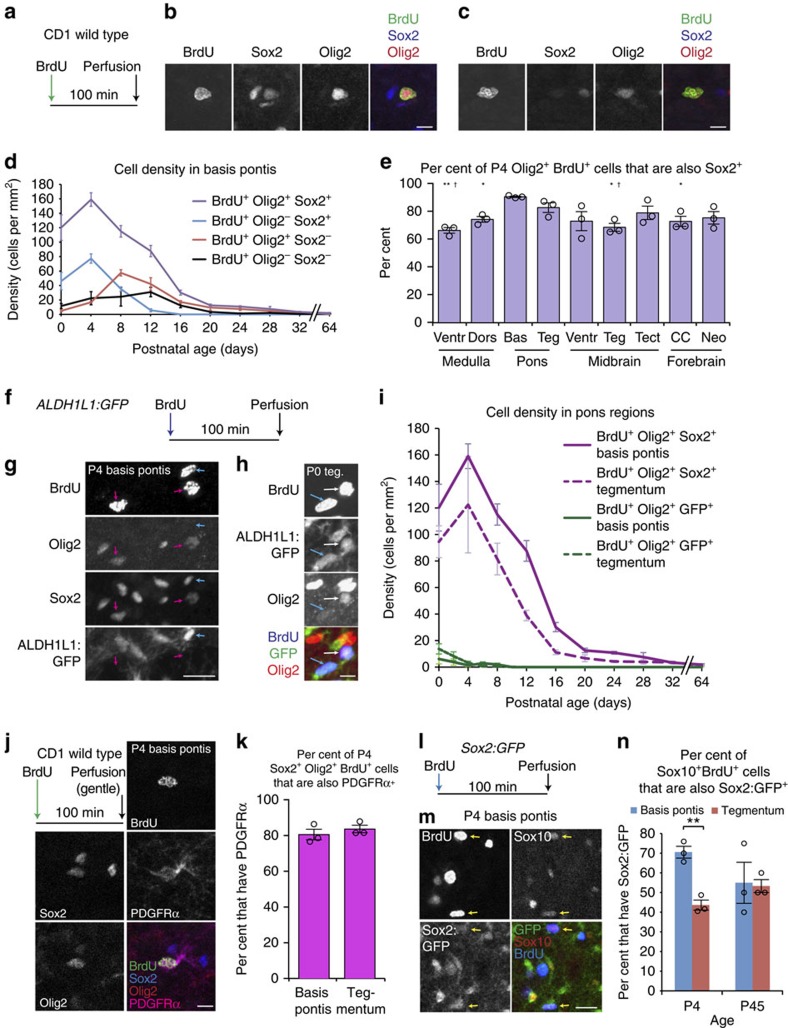
Sox2 is expressed in most postnatal pons OLPs. (**a**–**e**) CD1 wild-type mice received BrdU 100 min before perfusion. (**b**) Representative P4 Sox2^+^Olig2^+^BrdU^+^ cell. Scale bar, 10 μm. (**c**) Representative P16 Sox2^−^Olig2^+^BrdU^+^ cell. Scale bar, 10 μm. (**d**) Breakdown of basis pontis BrdU^+^ cell density by Sox2/Olig2 co-staining, through postnatal development; mean±s.e.m., *n*=3 mice per timepoint. Sox2^+^Olig2^+^BrdU^+^ cells outnumber any other cell type (*F*_1,40_≥196.91, *P*<0.0003, Bonferroni-adjusted two-way ANOVA based on cell type and age; tegmentum shown in Supplementary Fig. 7a). (**e**) P4 tissue from the indicated regions was analysed for the proportion, among total Olig2^+^BrdU^+^ cells, of Sox2^+^Olig2^+^BrdU^+^ triple-positive cells; *n*=3 mice, mean±s.e.m. Unpaired *t*-test confirmed Olig2^+^BrdU^+^ cells were more often Sox2^+^ in basis pontis than in ventral medulla (*P*=0.0055), dorsal medulla (*P*=0.0123), midbrain tegmentum (*P*=0.0152) or corpus callosum (*P*=0.0380), and more often Sox2^+^ in pontine tegmentum than ventral medulla (*P*=0.0231) or midbrain tegmentum (*P*=0.0366). *P<0.05 versus basis pontis; **P<0.01 versus basis pontis; ^†^*P*<0.05 versus pontine tegmentum. (**f**–**h**) *ALDH1L1:GFP* mice received BrdU 100 min before perfusion. (**g**) Sox2^+^Olig2^+^BrdU^+^ALDH1L1:GFP^−^ cells (pink arrows) and Sox2^+^ALDH1L1:GFP^+^BrdU^+^Olig2^−^ cells (blue arrow) in P4 basis pontis. Scale bar, 20 μm. (**h**) BrdU^+^ALDH1L1:GFP^+^Olig2^+^ (white arrow) and BrdU^+^ALDH1L1:GFP^+^Olig2^−^ (blue arrow) subtypes of proliferative astrocytes in P0 tegmentum. Scale bar, 10 μm. (**i**) Densities of GFP^+^Olig2^+^BrdU^+^ cells in *ALDH1L1:GFP* pons, and of Sox2^+^Olig2^+^BrdU^+^ cells in wild-type pons; mean±s.e.m., *n*=3 mice per strain per timepoint. (**j**,**k**) CD1 wild-type mice age P4 were given BrdU as in **a**, perfused with short postfix (100 min, 4 °C), cryosectioned, and co-stained as indicated. (**j**) Representative basis pontis OLP; scale bar, 10 μm. (**k**) PDGFRα was expressed in ∼80% of Sox2^+^Olig2^+^BrdU^+^ progenitors; *n*=3 mice, mean±s.e.m. (**l**–**n**) *Sox2:GFP* mice received BrdU 100 min before perfusion. (**m**) P4 pons tissue co-stained as indicated. Yellow arrows indicate representative BrdU^+^GFP^+^Sox10^+^ triple-positive cells. Scale bar, 15 μm. (**n**) *Sox2:GFP* pons regions aged P4 or P45 were analysed for the proportion, among proliferative OLPs (Sox10^+^BrdU^+^), of cells expressing Sox2:GFP; this percentage was greater in P4 basis pontis than tegmentum (*P*=0.0026, unpaired *t*-test); *n*=3 mice per timepoint, mean±s.e.m., **P<0.01.

We turned our attention to the parenchymal progenitors, the largest progenitor pool in the postnatal pons. Again using BrdU to label proliferative cells 100 min before perfusion, we used mice carrying cell type-specific reporter transgenes to determine the identity of proliferative cells (Fig. 7a). *NG2:DsRed* mice report DsRed in NG2-expressing cells, including OLPs and pericytes^36^, so we co-stained for Sox10 and identified OLPs as BrdU^+^DsRed^+^Sox10^+^ triple-positive cells (Fig. 7b). *ALDH1L1:GFP* mice report GFP in astrocytes^32^, and proliferative parenchymal astrocytes were vimentin^+^ as well (Fig. 7c). It was difficult to colocalize nuclear and filamentous markers, so we identified proliferative astrocytes as BrdU^+^ALDH1L1:GFP^+^ cells. Co-staining for Olig2 generally revealed segregation from astrocytes (Fig. 8g, Supplementary Fig. 7b); however, a small subpopulation of BrdU^+^ALDH1L1:GFP^+^Olig2^+^ astrocytes was also identified (Fig. 8h, Supplementary Fig. 7c). We measured proliferation among these cell types through postnatal development and found that proliferative OLPs outnumbered proliferative astrocytes at all ages from P4 onward (Fig. 7d). In P4 basis pontis, the densities of these cell types were 192.8±2.7 proliferative OLPs per mm^2^ and 73.0±3.9 proliferative astrocytes per mm^2^; in P4 tegmentum, 151.4±10.3 proliferative OLPs per mm^2^ and 12.4±2.9 proliferative astrocytes per mm^2^. Proliferation persisted to a later age in basis pontis than tegmentum for both cell types: proliferative OLPs in basis pontis showed a sustained peak density from P4 to P8, while OLP proliferation in tegmentum peaked at P4 and began to decline by P8; proliferative astrocytes plateaued from P0–P4 in basis pontis, but declined in tegmentum as soon as P4. OLP proliferation declined to <10% of its peak in both basis pontis and tegmentum by P24, while astrocyte proliferation decreased to <10% of its peak by P8 in tegmentum, and P12 in basis pontis. In adults (P64), we observed a small number of proliferative OLPs in both basis pontis (2.41±0.74 cells per mm^2^) and tegmentum (3.15±1.28 cells per mm^2^), comparable to the total proliferation in adult wild-type pons parenchyma (Fig. 3h); no astrocyte proliferation was observed after P12.

The predominance of OLPs at the P4–P8 proliferative peak, and the marked postnatal increase in OL number, raised the question of what proportion of OLPs are actively cycling. We therefore administered BrdU repeatedly to wild-type mice from P4.5–P8, and perfused mice 100 min after the last dose, at P8. We observed BrdU in 86.0%±2.6% of OLPs in basis pontis, and 63.2%±4.2% of OLPs in tegmentum (Supplementary Fig. 4g–i). This result echoes the denser OLP proliferation in basis pontis than tegmentum (Fig. 7d), suggests that nearly all basis pontis OLPs divided during the postnatal proliferative peak between P4–P8, and may in part account for the greater expansion of the oligodendroglial population in basis pontis compared with tegmentum (Fig. 4f).

Despite the finding that proliferative OLPs outnumber proliferative astrocytes, a pulse-chase experiment (Fig. 7e–h) with a single dose of BrdU at P4 yielded, among BrdU^+^ cells at P28, more astrocytes (60.7%±5.3%) than oligodendroglia (28.6%±4.0%) in basis pontis, and an even ratio of astrocytes to oligodendroglia in tegmentum (49.1%±4.7% versus 44.3%±2.4%). Taken together with our stereologic counts of oligodendroglia (Fig. 4), this result suggested two possibilities: (1) P4 OLPs may divide so many times by P28 that many of them dilute out their BrdU signal, and/or (2) oligodendroglia might undergo cell death. Consistent with the first possibility, BrdU staining was weaker and more punctate in P28 OLs than in P28 astrocytes (Fig. 7f,g). Further supporting the first possibility, oligodendroglia accounted for a lower per cent of P28 BrdU^+^ cells in basis pontis than in tegmentum (Fig. 7h). Supporting the second possibility, apoptosis has been documented in young OLs^37^, and in P12 basis pontis we observed Sox10^+^ nuclei surrounded by cytoplasmic, cleaved caspase-3 staining in an OL-like morphology (Supplementary Fig. 8e). We also investigated a third possibility, that P4 ‘OLPs' might produce astrocytes, by using *NG2CreER* mice^38^. *NG2CreER;Ai14;ALDH1L1:GFP* mice receiving tamoxifen at P0 or P4 did not show any TdTomato^+^ (TdT^+^) cells expressing *ALDH1L1:GFP* at P8, or any TdT^+^ cells expressing GFAP at P60, but instead showed TdT^+^ pericytes and Sox10^+^ or Olig2^+^APC^+^ cells (Supplementary Fig. 8a–d), consistent with forebrain^38^. Altogether, these experiments show that the postnatal pons has proliferative OLPs and astrocytes, pons OLPs are lineage restricted, and OLPs are the predominant proliferative population at all postnatal ages.

### Olig2 and Sox2 define proliferative progenitor subclasses

To further define the nature of proliferative pontine glial precursors, we used the markers Sox2 and Olig2, which can be expressed in tumour-propagating glioma cells^39^,^40^. Using wild-type mice, we delivered a single dose of BrdU 100 min before perfusion (Fig. 8a), and performed co-immunofluorescent labelling of BrdU, Olig2 and Sox2 (Fig. 8b–e, Supplementary Figs 7a and 9a,b). The largest pool of BrdU^+^ cells co-expressed Sox2 and Olig2 (Fig. 8b,d; Supplementary Fig. 7a). During the P4 proliferative peak, the proportion of BrdU^+^Olig2^+^ cells coexpressing Sox2 was greater in basis pontis than in ventral or dorsal medulla, midbrain tegmentum, or even corpus callosum; and likewise a greater proportion co-expressed Sox2 in pontine tegmentum than in midbrain tegmentum (Fig. 8e). Thus the postnatal mouse pons was particularly enriched in Sox2^+^Olig2^+^BrdU^+^ progenitors. The remaining progenitors included a Sox2^+^Olig2^−^BrdU^+^ population peaking at P4, a Sox2^−^Olig2^+^BrdU^+^ population (Fig. 8c,d, Supplementary Fig. 7a) peaking at P8, and a small Sox2^−^Olig2^−^BrdU^+^ population most of which lined blood vessels and had elongated nuclei consistent with endothelial cells. The Sox2^+^Olig2^−^BrdU^+^ population likely corresponds to proliferative astrocytes, as immunostaining revealed broad Sox2 expression in ALDH1L1:GFP^+^ cells (Supplementary Fig. 9c), and as its numbers (Fig. 8d) were comparable to ALDH1L1:GFP^+^BrdU^+^ cells (Fig. 7d, Supplementary Fig. 7c).

The Sox2^+^Olig2^+^BrdU^+^ cells were predominantly not astrocytes, because in *ALDH1L1:GFP* mouse pons, Sox2^+^Olig2^+^BrdU^+^ cells were usually GFP^−^ (Fig. 8f,g; Supplementary Fig. 9b). While a subpopulation of BrdU^+^ ALDH1L1:GFP^+^ astrocytes expressed Olig2 (Fig. 8h), such cells were sparse compared with the BrdU^+^Olig2^+^Sox2^+^ progenitors measured in wild-type mouse pons (Fig. 8i, Supplementary Fig. 7c). The total Olig2^+^BrdU^+^ population, measured as the sum of Sox2^+^Olig2^+^BrdU^+^ and Sox2^−^Olig2^+^BrdU^+^ populations, showed similar numbers to committed OLPs (Fig. 7d): in basis pontis, a sustained peak from P4 (176±10 cells per mm^2^) to P8 (173±4 cells per mm^2^); in tegmentum, a peak at P4 (147±40 cells per mm^2^). And remarkably, at P4, nearly all BrdU^+^Olig2^+^ cells co-expressed Sox2: 90.4%±0.4% in basis pontis and 82.6%±3.5% in tegmentum (Fig. 8e). Altogether, this suggested that the committed OLP population included many Sox2^+^Olig2^+^ cells. Sox2 was long considered a marker of stem cells, but has recently been documented in embryonic OLPs of forebrain and spinal cord^41^. We therefore performed immunostaining in P4 *ALDH1L1:GFP* tissue to test whether pontine Sox2^+^Olig2^+^ cells co-expressed the canonical OLP marker PDGFRα, or the astrocyte reporter ALDH1L1:GFP (PDGFRα and ALDH1L1:GFP were 99% mutually exclusive in pons, Supplementary Fig. 9f). Remarkably, 89.8%±1.7% of Sox2^+^Olig2^+^ cells in basis pontis were PDGFRα^+^ALDH1L1:GFP^−^ (Supplementary Fig. 9d–f). NG2 protein was similarly localized to Sox2^+^Olig2^+^ALDH1L1:GFP^−^ cells (Supplementary Fig. 9g). Next, examining CD1 wild-type mice, we found PDGFRα co-expressed in 80.5%±3.0% of basis pontis Sox2^+^Olig2^+^BrdU^+^ cells, and in 83.6%±2.1% of tegmentum Sox2^+^Olig2^+^BrdU^+^ cells (Fig. 8j,k). To test Sox2 coexpression with a third marker of committed oligodendroglia, Sox10, we obtained mice carrying a *Sox2:GFP* knock-in allele^42^. We performed a single injection of BrdU 100 min before perfusion at P4 or P45 (Fig. 8l), and co-stained pons sections for BrdU, GFP, and the oligo-lineage marker Sox10 (Fig. 8m). We found that 70.5%±3.0% of Sox10^+^BrdU^+^ cells co-expressed Sox2:GFP in the P4 basis pontis, and 43.7%±2.6% in P4 tegmentum, while Sox10^+^BrdU^+^ cells in both pons regions showed roughly 50% Sox2:GFP expression at P45 (Fig. 8n). Altogether, these results indicate that the postnatal pons is rich with Sox2^+^Olig2^+^ proliferative OLPs, and also contains a smaller, later-peaking, Sox2^−^Olig2^+^ proliferative population.

### Postnatal Sox2^+^ cells produce over 90% of adult pons OLs

**Figure 9:**
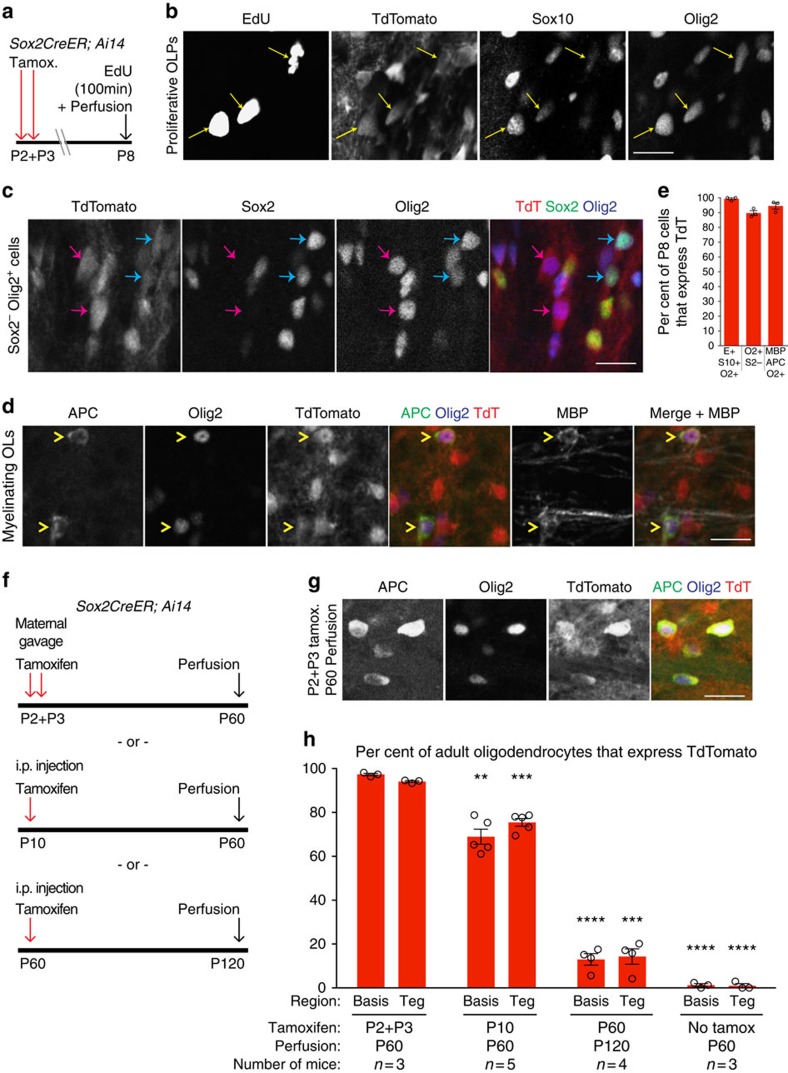
Postnatal Sox2^+^ progenitors give rise to ∼95% of adult pons OLs. (**a**–**e**) Short-term *Sox2CreER* fate mapping. *Sox2CreER;Ai14* pups were given tamoxifen (by maternal gavage) at age P2 and P3, and EdU intraperitoneally at age P8, 100 min before perfusion. (**b**–**d**) TdT^+^Olig2^+^ cell types observed in P8 basis pontis; scale bars=20 μm. (**b**) TdT^+^Olig2^+^EdU^+^Sox10^+^ proliferative OLPs (arrows). (**c**) TdT^+^Olig2^+^Sox2^+^ (cyan arrows) and TdT^+^Olig2^+^Sox2^−^ cells (magenta arrows). (**d**) TdT^+^Olig2^+^APC^+^MBP^+^ myelinating OLs (arrowheads). (**e**) Quantitation of the proportion of each P8 cell type shown in (**b**–**d**) that coexpress TdT, demonstrating origin from postnatal Sox2^+^ progenitors. Data shown are from basis pontis. ‘E'=EdU, ‘S10'=Sox10, ‘S2'=Sox2, ‘O2'=Olig2. Graph shows individual replicates with mean±s.e.m. of *n*=3 mice. (**f**–**h**) Long-term *Sox2CreER;AI14* fate mapping. Tamoxifen induction was performed in one cohort by maternal gavage at P2 and P3, and in a second cohort by IP injection at P10; these two cohorts were perfused at P60. Another cohort received tamoxifen at P60 and was perfused at P120. (**g**) TdT^+^Olig2^+^APC^+^ mature OLs in adult basis pontis of P2–P3 tamoxifen-induced *Sox2CreER;Ai14* mice; scale bar, 20 μm. (**h**) Quantitation (by pons region and tamoxifen timing) of the proportion of mature OLs (APC^+^Olig2^+^) that coexpress TdT, demonstrating origin from Sox2^+^ progenitors at the indicated postnatal ages. A cohort of no-tamoxifen control mice showed TdT expression in∼1% of adult pons OLs. Graph shows individual replicates with mean±s.e.m. of the indicated *n* mice. **P<0.01, ***P<0.001, ****P<0.0001 (unpaired *t*-test, versus the corresponding region of P2–P3 dosed mice).

The presence and timing of Sox2^+^ and Sox2^−^ subpopulations of proliferative Olig2^+^ cells suggested a possible lineage progression of Sox2^+^Olig2^+^, to Sox2^−^Olig2^+^, to myelinating oligodendrocytes. To test whether postnatal Sox2^+^ cells produced Sox2^−^Olig2^+^ cells and mature OLs, we used *Sox2CreER*;*Ai14* reporter mice. We delivered tamoxifen by maternal gavage at P2–P3, followed by a single dose of EdU 100 min before perfusion at P8 (Fig. 9a). We found that in basis pontis, TdT^+^ cells accounted for: 99.3%±0.7% of Olig2^+^Sox10^+^EdU^+^ proliferative OLPs (Fig. 9b,e); 97.8%±2.2% of Olig2^+^Sox2^+^ cells and 89.7%±1.6% of Olig2^+^Sox2^−^ cells (Fig. 9c,e); and 94.5%±2.1% of APC^+^Olig2^+^MBP^+^ myelinating OLs (Fig. 9d,e). These results support the hypothesis that postnatal Sox2^+^ progenitors generate all stages of the oligodendrocyte lineage. To determine the extent to which postnatal Sox2^+^ progenitors contributed oligodendrocytes to the mature pons, we delivered tamoxifen to *Sox2CreER;Ai14* mice at P2–P3, P10, or P60 (Fig. 9f), perfused 50–60 days later, and co-stained for TdT, Olig2, and APC. Tamoxifen treatment at P2+P3 led to TdT expression in 97.3%±0.5% of APC^+^Olig2^+^ OLs in P60 basis pontis, and in 94.1%±0.4% of OLs in P60 tegmentum (Fig. 9g,h). Delivery of tamoxifen at P10 labelled 69.0%±3.4% of P60 basis pontis OLs and 75.5%±1.8% of P60 tegmentum OLs. Delivery of tamoxifen to P60 mice labelled only 13.0%±2.6% of P120 basis pontis OLs (Fig. 9h) and 14.3%±4.3% of P120 tegmentum OLs. This is consistent with the lower level of proliferative Sox2^+^Olig2^+^ cells in adult pons (Fig. 8d). A no-tamoxifen control showed TdT expression in only 1.0–1.2% of P60 pons OLs (Fig. 9h), indicating low spontaneous background recombination.

The Sonic Hedgehog pathway drives embryonic oligodendrogenesis^24^,^43^; its effector Gli1 has been previously proposed as a postnatal pons progenitor cell marker^5^, and postnatal Gli1^+^ forebrain progenitors are known to produce OLs^44^. We therefore tested if pons OLs were derived from Gli1-expressing progenitors. We found that postnatal tamoxifen treatment at P2–3 or P10 in *Gli1CreER;Ai14* mice^45^ yielded no TdT^+^Olig2^+^APC^+^ OLs in adult pons, but sparse TdT^+^ astrocytes positive for S100β and/or GFAP (Supplementary Fig. 10). Thus Gli1-expressing cells at P2–3 or P10 do not contribute to postnatal pontine oligodendrogenesis, by contrast with Sox2^+^ cells. Tamoxifen-treated *Sox2CreER;Ai14* mouse pons also contained TdT^+^ astrocytes (Supplementary Fig. 10c), reflecting pontine astrocytes' common Sox2 expression (Supplementary Fig. 9c). In addition to that expected result, our fate mapping experiments demonstrate that postnatal Sox2^+^ progenitors populate the adult pons with ∼95% of its oligodendrocytes.

## Discussion

We have shown here that, as in humans^3^, the mouse pons grows markedly after birth, driven by a single wave of proliferation. We identified Sox2^+^ Olig2^+^ parenchymal progenitors as the main proliferative progenitor population, and found that postnatal Sox2^+^ cells give rise to nearly all adult pons OLs.

The postnatal mouse basis pontis quintupled and the tegmentum quadrupled in size, placing the pons among the brain regions with the most pronounced postnatal growth—comparable to that of cortex and hippocampus, but less than that of cerebellum^27^,^28^. As in humans^3^, the mouse basis pontis experienced more rapid growth and greater proliferation than the tegmentum. There were also interesting differences between human and mouse pons: at birth, the human basis pontis already contains thin myelinated fibres, and proliferation declines after birth; however, the mouse basis pontis experiences its onset of myelination and decline in proliferation during the second postnatal week. The P4 proliferative peak may therefore correspond to a pre-term peak proliferative stage in human pons, which has not yet been studied.

We found that postnatal pontine growth did not involve the addition of new neurons, but rather correlates with a dramatic increase in the oligodendroglial lineage. The basis pontis at P16 contained 18 times as many oligodendroglia as at P0, and the tegmentum at P16 contained 10 times as many as at P0, suggesting that basis pontis OLPs undergo at least four rounds of postnatal division and tegmentum OLPs undergo at least three. However, the expansion might be carried out by subpopulations dividing five times or more. It is also possible that the population expansion is even greater than observed, but appears reduced due to cell death^37^ or migration out of pons.

We defined three compartments of postnatal progenitor cell proliferation in the pons: VZ, midline and parenchyma. The VZ and midline contributed only a few cells, generally close to these proliferative domains. The parenchymal compartment generated the majority of new cells; some progenitors were proliferative astrocytes, but a greater number corresponded to proliferative OLPs. BrdU^+^Sox2^+^Olig2^+^ OLPs were the largest parenchymal progenitor pool, and postnatal Sox2^+^ progenitors produced ∼95% of adult pons oligodendrocytes. These observations suggest that Sox2^+^ progenitors play a key role in postnatal oligodendrogenesis. A prior study suggested that Sox2 acts in embryonic spinal cord oligodendroglia to block maturation and myelination during the OL stage^41^; however, we observed that a sizeable subset of postnatal pontine Olig2^+^ cells downregulate Sox2 while still proliferating, that is, as OLPs. We found a small subpopulation of Sox2^+^Olig2^+^ progenitors that continued to proliferate in adult mouse pons. Interestingly, Sox2^+^Olig2^+^ cells have also been described in adult human white matter^46^. Our *Sox2CreER* fate mapping demonstrates that adult Sox2^+^ cells continue to generate oligodendrocytes *in vivo*.

The functions of sustained postnatal pontine growth and oligodendrogenesis remain unknown. Oligodendrogenesis is regulated by neuronal activity^47^,^48^, so the late formation of oligodendrocytes may be linked to postnatal neural activity flowing through the pons. By delaying the onset of myelination, the pons might allow experience to guide which circuits become myelinated. It is appealing to speculate that the maturation of cells in the basis pontis is developmentally coordinated with the maturation of cerebellar target regions, whose growth is mostly postnatal. Interestingly, the middle cerebellar peduncle is among the last tracts in the brain to be myelinated^27^. Given the prominence of motor pathways in the pons, sustained postnatal oligodendrogenesis may allow the development of high-speed circuit conductances in an experience-dependent manner during a critical period of acquisition of motor coordination. Similarly, experience-dependent circuit enhancement could occur in the autonomic regions of the growing postnatal tegmentum that undergo later myelination.

Tumour incidence may be correlated with a tissue's normal developmental proliferative activity^49^. Our findings may partly explain the pons's susceptibility to tumour formation. The pons is the site where Diffuse Intrinsic Pontine Glioma (DIPG), the most lethal paediatric CNS tumour^4^, appears. It is suspected that DIPGs have a progenitor cell origin^50^. This progenitor's identity remains uncertain. OLPs have been shown as a cell of origin for some paediatric forebrain gliomas^51^,^52^ and adult gliomas^53^,^54^. DIPGs frequently express Olig2 and Sox2 (ref. 55); our finding of PDGFRα coexpression in the vast majority of P4 BrdU^+^Olig2^+^Sox2^+^ cells is notable given that *PDGFRA* is commonly amplified in DIPG^55^,^56^. Most DIPGs also contain a mutation in Histone H3 at lysine 27 (refs 57, 58), which might epigenetically trap cells in a progenitor state^50^ and prevent repression of Olig2 or Sox2; one study showed that a H3K27M DIPG patient tumour-derived cell line had increased Olig2 expression correlated with decreased H3K27 trimethylation at the *OLIG2* locus, when compared with mouse forebrain-derived neurospheres^59^. All this suggests that the BrdU^+^Olig2^+^Sox2^+^ OLPs, which peak at P4 and are enriched in basis pontis, are a prime candidate for DIPG cell of origin. However, despite the ventral preference for growth and proliferation (present study and ref. 3) and the propensity of DIPG to spread ventrally^60^, we cannot rule out that some DIPGs might originate dorsally. We observed significant OLP proliferation in the tegmentum at slightly earlier stages than basis pontis; interestingly, a fraction of DIPGs express the dorsal marker Pax3 (ref. 61). We also cannot exclude that DIPG might originate from astrocytes. Astrocytes accounted for ∼¼ of proliferation in P4 basis pontis; all astrocytes express Sox2, and a subpopulation coexpress Olig2. And finally, it is possible that DIPG may originate prenatally. In one study on embryonic oligodendrogenesis, it was shown that rhombomere 4, within the pons, produces more oligodendroglia than neighbouring segments^26^. In light of our postnatal observations, the embryonic pattern of OLP production^26^ may prepare the pons for its dramatic postnatal growth, and may render the pons rich in potentially gliomagenic progenitors.

The typical presentation of DIPG at 5–9 years led one group to hypothesize a ‘second peak' of progenitor cells in ‘middle childhood'^5^. However, when we examined the postnatal pons's normal course of proliferation, we found only a single peak: at birth in human^3^ and at P4 in mouse (present study). Furthermore, a single proliferative peak was evident for each progenitor cell population. While we cannot exclude that a tumour might arise from the few proliferative cells that persist to later ages, proliferation data favour an origin for DIPG during the single, early-childhood proliferative peak, from Sox2^+^Olig2^+^ progenitors.

This study demonstrates that the postnatal mouse pons undergoes a dramatic increase in size. We have identified proliferative oligodendroglia and astroglia in postnatal pons parenchyma, shown that pons OLPs have early Sox2^+^ and later Sox2^−^ stages, and demonstrated that ∼95% of pons oligodendrocytes derive from postnatal Sox2^+^ progenitors. This dramatic pontine cell addition may be linked to the acquisition of key developmental milestones in children, and may predispose the pons to tumour formation. The progenitor cells identified here represent candidates for targeting in future tumour models.

## Methods

### Animals

All animal procedures were performed in accordance with NIH guidelines. Animal protocols were approved and supervised by the UCSF IACUC. Transgenic strains, uses and sources are listed in Supplementary Fig. 1. Transgenic mice were on a mixed background containing CD1 and C57BL/6. ‘Wild type' animals in all figures were CD1 (Charles River, Wilmington, MA), except for Fig. 7e–h in which ‘wild type' represents GFP-negative littermates of the *ALDH1L1:GFP* mice used elsewhere in those panels. Mice of both sexes were used. The only species used was *Mus musculus*. BrdU (Sigma-Aldrich, St Louis, MO) was dissolved in sterile phosphate-buffered saline (PBS) and delivered by intraperitoneal injection at a dose of 50 mg kg^−1^; EdU (Invitrogen, Waltham, MA) was prepared identically and delivered at an equimolar dose, 41 mg kg^−1^. Tamoxifen was dissolved in sterile-filtered corn oil and delivered by maternal gavage at 5 mg per day, or intraperitoneally into mice P10 or older (4 mg per 30 g body weight). Neonatal intracerebroventricular viral injections were performed in P1 mice using the following stereotaxic coordinates: (2.0 mm posterior from intraocular line, 0.8 mm lateral from midline, 1.5 mm deep from skin), with a microinjection needle positioned vertically (0 degrees). 1 μl of virus (Adeno-CMV-Cre, Vector Biolabs, 10^10^ p.f.u. ml^−1^; RCAS-GFP, ∼2 × 10^7^ p.f.u. ml^−1^) was delivered into lateral ventricle. Direct injection into the fourth ventricle could have risked accidental puncture of the ventricular surface and labelling of pons parenchyma, as well as inadvertent contact with cerebellar mossy fibre axons from the precerebellar pontine nuclei. Transcardial perfusion was performed as previously described^62^; most samples underwent post-fixation overnight at 4 °C, but for samples used in PDGFRalpha and NG2 antibody staining (Fig. 8j,k, Supplementary Fig. 8d–g), post-fixation was limited to 100 min at 4 °C so as to preserve antigenicity.

### Histology

Because of the placement of the mesencephalic and pontine flexures, a standard coronal section^63^,^64^ cannot contain the full dorsal–ventral extent of the pons, from basis pontis through tegmentum up to the 4th ventricle. Therefore, for many experiments (including parts or all of Figs 1, 2, 4 and 6, and Supplementary Fig. 7) we blocked tissue in an oblique plane, orthogonal to the spinal cord, illustrated in Fig. 1a. This plane contains basis pontis, tegmentum and fourth ventricle, allowing measurement of pons size and comparisons of basis pontis and tegmentum in the same section. For studies of proliferation and/or the ventricular zone, we blocked sections in the standard coronal plane, allowing a wider ventricular space to separate brainstem from cerebellum. For viral fate mapping following lateral ventricle injection (Fig. 5 and Supplementary Fig. 6), sections were coronal or sagittal. For all experiments except PDGFRalpha and NG2 protein staining, tissue sections were prepared at 50 μm thickness on a sliding-freezing microtome (Leica Microsystems, Buffalo Grove, IL), following cryoprotection in 30% sucrose in PBS+0.1% sodium azide, and subjected to immunohistochemistry as floating sections. For PDGFRalpha and NG2 protein staining, tissue sections were prepared at 30 μm thickness on a Cryostat (Leica), following cryoprotection as above, embedding in O.C.T. (Sakura, Torrance, CA), and frozen storage at −80 °C; tissue was collected on SuperFrost Plus slides (Fisher Scientific, Hampton, NH) and subjected to immunohistochemistry on the slides. Some stains required antigen retrieval in 10 mM citrate buffer (pH6.0) at 95 °C for 10–20 min or 0.3 M glycine buffer (pH7.4) at 56 °C for 45 min. All sections were blocked for 30–60 min at room temperature in buffer containing PBS, 0.1% Triton X-100 (Fisher Scientific), and 2–5% species-specific serum (depending on host species of secondary antibody); antibodies were diluted in blocking buffer for primary antibody incubation (overnight at 4 °C) and secondary antibody incubation (2 h at room temperature); rinses were performed in PBS/0.1% Triton. Primary antibodies were applied simultaneously, except for anti-BrdU (described below).

### Antibodies

Primary antibodies and dilutions are described in Supplementary Fig. 2. Secondary antibodies were from Invitrogen or Jackson Immunoresearch (West Grove, PA), raised in donkey (if one primary antibody in the co-stain was raised in goat) or in goat (in all other cases). IgG subclass-specific secondary antibodies were used where available; secondary antibodies were conjugated to Alexa Fluor 488, 546, 555, 568, 594 or 647 dyes (Invitrogen), or to DyLight 488, Cy3 or Cy5 dye (Jackson Immunoresearch), and all secondary antibodies were used at 1:750 dilution. DAPI (1:5,000; Sigma) was added during secondary antibody incubation; in samples that would subsequently be immunostained for BrdU, DAPI was deferred till the final secondary antibody incubation.

### Thymidine analogue detection

For BrdU co-staining, a sequential protocol was used in which non-BrdU antigens were labelled by primary and secondary antibodies as above; then, fluorophores were fixed into tissue by 15 min in 4%PFA, followed by denaturation with 2 N HCl at 37 °C and quenching in boric acid buffer pH8.5 as previously described^31^, and finally, blocking and immunostaining with anti-BrdU primary and fluorophore-conjugated anti-rat secondary antibodies. For EdU detection, the Click-iT EdU Alexa Fluor 647 kit (Invitrogen) was used before immunostaining according to manufacturer's instructions. Control stains were performed to verify that BrdU and EdU methods labelled cells in a distribution comparable to Ki67 immunostaining.

### Imaging

Tiled images of entire sections were collected on a Zeiss Axiovert 200 M inverted epifluorescence microscope under × 5 or × 10 objectives (Carl Zeiss, Pleasanton, CA), using the ‘Virtual Tissue' module of NeuroLucida/StereoInvestigator software (MicroBrightField, Williston, VT), an automated stage controller (MicroBrightField), and an AxioCam (Carl Zeiss) or Hamamatsu Orca camera (Hamamatsu, Shizuoka, Japan). High-resolution images were acquired on SP5 Confocal Microscopes (Leica), equipped with LAS AF software, under a × 20 objective and optical zoom to resolutions between 0.757 μm px^−1^ and 0.278 μm px^−1^. Fluorophores were excited by sequential scans using fixed-wavelength or white-light lasers, with emission detection windows chosen to avoid bleed-through in single-fluorophore controls. Individual image channels were merged into colour images in batches using CellProfiler software^65^, or for few-image experiments, manually using Adobe Photoshop (Adobe Systems, San Jose, CA). All images shown are representative of *n*≥3 mice.

### Size measurements

DAPI-stained mouse pons sections, oriented in the oblique plane described above, were collected as tiled images in NeuroLucida as described, then pons regions were measured offline in NeuroLucida software by drawing contours around basis pontis and whole pons. Basis pontis was defined as ‘pons proper'^66^, that is, pontine grey nuclei plus adjacent white matter tracts (transverse fasciculus pontis, longitudinal fasciculus pontis, and middle cerebellar peduncle in its ventralmost aspect, that is, excluding portion contacting tegmental nuclei). Pontine tegmentum was defined by subtraction of basis pontis from whole pons. Volumes for basis pontis and tegmentum were computed as the sum of respective regional areas in every second section through pons, times the distance between sections (100 μm=50 μm section thickness × every second section). For the size study, we used CD1 wild-type mice at ages P0 (*n*=3), P4 (*n*=4), P10 (*n*=3), P16 (*n*=3), P24(*n*=3) and P64 (adult, *n*=3).

### Tracing of myelinated tracts

The tracing in Fig. 2a, showing major white matter tracts in mouse pons, was obtained by offline NeuroLucida tracing of a MBP-stained P16 pons section in the oblique orientation. Tracing was exported as a PostScript file and imported as vector art into Adobe Illustrator (Adobe Systems).

### Proliferation maps

Tiled images of mouse brainstem sections, oriented in coronal plane to better distinguish VZ of 4th ventricle, were co-stained for BrdU, Vimentin, and GFAP, then imaged in StereoInvestigator software and traced offline. Markers were placed at the location of every BrdU^+^ nucleus, with distinct markers for cells in VZ, cells contacting Vimentin^+^ processes along the midline, cells contacting GFAP^+^ processes branching off the midline, and cells in parenchyma. Maps were exported as postscript files and imported as vector art into Adobe Illustrator.

### Proliferation measurements

Mouse brainstem sections were immunostained for BrdU and other markers as described above. For VZ proliferation (Fig. 3f), we used StereoInvestigator to perform live counting under a × 10 objective, identifying BrdU^+^ cells in VZ by their overlap with the dense DAPI zone along the fourth ventricle; ventricular surface length was also measured live in StereoInvestigator. We analysed every third section (in ages P0–P12) or every sixth section (in ages P16 and up) through the pontine VZ. Density was computed as the ratio of BrdU^+^ VZ cells to VZ length. For measurements of proliferation density among parenchymal populations (Figs 3, 7 and 8; Supplementary Figs 3 and 7), counting was done using confocal images acquired as overlapping individual fields at 0.505 μm px^−1^ resolution spanning the entire (lateral-medial-lateral) extent of basis pontis or pontine tegmentum; we used StereoInvestigator for manual alignment of individual fields, region-area measurements and counting of BrdU^+^ cells and colocalized markers (Sox2, Olig2, GFP, Sox10 and DsRed). For pons parenchyma, proliferative cell density was computed as the ratio of BrdU^+^ cells to area of basis or pontine tegmentum. For the pontine midline domain, which was defined as the medial white matter space in the section and at all ages was within 50 μm of the midline, proliferative cell density was defined as the ratio of BrdU^+^ cells to length of midline. For midbrain, medulla and forebrain regions, proliferation density was measured using a single confocal field per animal, taken at the following sites: superior colliculus (midbrain tectum), dorsal raphe nucleus (midbrain tegmentum), interpeduncular nucleus (ventral midbrain), prepositus hypoglossal nucleus (dorsal medulla), inferior olivary nucleus (ventral medulla) and dorsal to the anterior horn of the lateral ventricle (corpus callosum and neocortex, both imaged in the same section). We used *n*=3 animals per timepoint, and up to 10 timepoints per strain: wild-type and *NG2:DsRed* mice were aged at 4-day intervals from P0–P32, plus P64 (adult); *ALDH1L1:GFP* mice were aged at 2-day intervals from P0–P12, plus P16, P20 and P64 (adult). For quantitation of proliferative OLPs in Sox2CreER fatemap (Fig. 9e), counts of P8 EdU^+^Olig2^+^Sox10^+^ cells were performed similarly in basis pontis, and the percentage of those cells coexpressing TdTomato (TdT) was reported.

### BrdU fatemap counts

BrdU labelled cells in the pulse-chase experiment (Fig. 7e–h) were imaged and counted offline in basis pontis and tegmentum as in acute proliferation experiments (described above). Astrocyte counts were from *ALDH1L1:GFP* mice and included cells that were ALDH1L1:GFP^+^BrdU^+^ and/or GFAP^+^BrdU^+^. Oligodendroglia counts were from wild-type (GFP^−^) littermates of the GFP^+^ mice and included cells that were Olig^+^BrdU^+^ and/or APC^+^BrdU^+^. *n*=3 mice were used per strain.

### Percentage counts and non-proliferative cell density counts

For measurements of the Sox2:GFP^+^ subpopulation of Sox10^+^BrdU^+^ cells at P45 (in Fig. 8l), overlapping confocal fields spanning the basis pontis or pontine tegmentum were acquired from one section per animal at resolution of 0.505 μm px^−1^; image alignment and exhaustive counting were performed offline in StereoInvestigator. For P4, the same procedure was performed, except due to the abundant proliferation, cells were selected by systematic uniform random sampling using the Area Fractionator probe and an area sampling fraction of 1/16. *n*=3 animals were used per timepoint. The fidelity of the *Sox2:GFP* reporter, previously demonstrated in ref. 42, was confirmed in pons by control co-immunostain of GFP and Sox2 protein. Density counts for the PDGFRα/ALDH1L1:GFP/Sox2/Olig2 costain (Supplementary Fig. 9f) were performed using the same sampling fraction and *n*, and a single confocal field for each region. For quantitation of PDGFRα coexpression among Sox2^+^Olig2^+^BrdU^+^ cells (Fig. 8k), exhaustive counts were performed from a single confocal field per animal in each of basis pontis (encompassing midline, transverse fasciculus pontis, pontine nuclei and corticospinal tract), tegmentum (adjacent to fourth ventricle) and neocortex (dorsal to anterior horn of lateral ventricle). For counts of non-proliferative cells in short-term *Sox2CreER;Ai14* fate mapping (Fig. 9e), systematic uniform random sampling within basis pontis was similarly performed offline, using confocal images from *n*=3 animals and an area sampling fraction of 1/16; average numbers of cells assessed for TdT positivity per animal were 90 myelinating OLs (range 65 to 123) and 83 cells positive for Olig2 and/or Sox2 (range 67–104). For measurements of the TdT^+^ percentage of APC^+^Olig2^+^ oligodendrocytes in adult *Sox2CreER;Ai14* mice (Fig. 9g), systematic uniform random sampling through basis pontis or tegmentum was performed live under a × 20 objective using the Optical Fractionator probe; the number of OLs counted per region per animal averaged 193, with minimum of 106 and maximum of 382. *n*≥3 animals were used per cohort, with specific *n* indicated in the figure.

### Stereologic counts

For counts of oligodendroglia, we performed co-immunofluorescent labelling of Olig2, Sox10, and APC, followed by live systematic uniform random sampling under a 20x objective using the Optical Fractionator probe in StereoInvestigator. OLs expressed APC plus at least one of Olig2 and Sox10; OLPs expressed both Olig2 and Sox10 but not APC. Thus a cell was only considered oligodendroglial if it expressed at least two of the three markers. For all samples the counting frame was 50 × 50 μm, and cell nuclei were used to define cell location with respect to the optical disector. Average mounted section thickness varied with age from 30 to 50 μm. Because of pons growth over postnatal development, and also because of the 5x size difference between mouse tegmentum and basis pontis, we chose the sampling parameters on a per-region, per-animal basis, to count an average of at least 200 cells per region per animal. Grid step size varied from 200 × 200 μm (at P0) to 350 × 350 μm (at P64) in basis pontis, and from 480 μm (at P0) to 800 μm (at P64) in pontine tegmentum; section interval varied from 4 (at P0) to 12 (at P64). The resulting number of oligodendroglia counted per basis pontis averaged 204, with a minimum of 83 and maximum of 305; the number of oligodendroglia counted per tegmentum averaged 242, with a minimum of 127 and maximum of 378. Stereologic counts of OLs and OLPs in basis pontis and tegmentum were obtained from three separate animals per timepoint, and mean counts and standard errors were computed from those three replicates.

### Statistics

Means, standard errors, and *P*-values (by two-tailed unpaired *t*-test) were computed in Microsoft Excel (Microsoft, Redmond, WA). Data were graphed in Excel or GraphPad Prism (GraphPad, La Jolla, CA). Three-way and two-way analysis of variance (ANOVA) was performed in Stata 13 (StataCorp, College Station, TX). Bonferroni correction was performed for multiple pairwise ANOVA comparisons of proliferation among regions (Fig. 3, Supplementary Fig. 3) and cell types (Fig. 8, Supplementary Fig. 6). For the three-way ANOVA in Fig. 7d, comparing proliferative cell density across ages, regions, and cell types (ALDH1L1:GFP^+^BrdU^+^ astrocytes versus Sox10^+^NG2:DsRed^+^BrdU^+^ OLPs), comparison was limited to the 7 timepoints measured in both strains (P0, P4, P8, P12, P16, P20, P64); the three additional timepoints uniquely measured in each strain (P2, P6, and P10 in *ALDH1L1:GFP*; P24, P28, and P32 in *NG2:DsRed*) were omitted from that calculation. A separate ANOVA was performed pooling single-strain timepoints with both-strain timepoints, in six groups: P0–P2, P4, P6–P10, P12, P16, P20–P32, and P64; *P*-values from this second ANOVA were as small as or smaller than those produced by the first. Therefore we reported the first, more conservative result. For all experiments, a minimum *n* of three mice per timepoint was chosen based not on power analysis but on past studies of brain region growth^28^ and hindbrain development^26^. The obvious differences in tissue size throughout postnatal development mooted attempts at blinding to sample identity. Distributions were assumed to be normal but this was not formally tested. No data points were excluded from analysis.

### Data availability

The data that support the findings of this study are available from the corresponding author on request.

## Additional information

**How to cite this article:** Lindquist, R. A. *et al.* Identification of proliferative progenitors associated with prominent postnatal growth of the pons. *Nat. Commun.* 7:11628 doi: 10.1038/ncomms11628 (2016).

## Supplementary Material

Supplementary Information Supplementary Figures 1-10
