# Supplementary material for: Identification of proliferative progenitors associated with prominent postnatal growth of the pons
Source: Nat Commun. 2016 May 18;7:11628. doi: 10.1038/ncomms11628 (PMC4873968; doi:10.1038/ncomms11628)
Supplement: Supplementary Information — Supplementary Figures 1-10 [file ncomms11628-s1.pdf]

Strain	Type of allele	Purpose	Source	Citation for allele
Ai14	Conditional reporter	TdTomato labeling of Cre-recombined cells and progeny (using viral or hereditary Cre)	Jackson Lab	Madisen *et al*., 2010 (PMID: 20023653)
ALDH1L1:GFP	BAC transgenic	Labeling of astrocytes	MMRRC/GENSAT	Cahoy *et al*., 2008 (PMID: 18171944)
FoxA2CreER	Knock-in	Fate-mapping of midline cells	Ross Metzger, UCSF	Park *et al*., 2008 (PMID: 18161057)
Gli1CreER	BAC transgenic	Fate-mapping of Gli1$^+$ cells	Alexandra Joyner, Memorial Sloan-Kettering	Ahn and Joyner, 2004 (PMID: 15315762)
Nestin:Tva	Transgene	RCAS retroviral fatemapping of VZ progenitors	Jackson Lab	Holland *et al*., 1998 (PMID: 9851974)
NG2:DsRed	BAC transgenic	Labeling of NG2$^+$ cells (OLPs and pericytes)	Lily Jan, UCSF	Ziskin *et al*., 2007 (PMID: 17293857)
NG2CreER	BAC transgenic	Fate-mapping of NG2$^+$ cells	Jackson Lab	Zhu *et al*., 2011 (PMID: 21266410)
Sox2:GFP	Knock-in	Labeling of Sox2$^+$ cells	Jackson Lab	Arnold *et al*., 2011 (PMID: 21982232)
Sox2CreER	Knock-in	Fate-mapping of Sox2$^+$ cells	Jackson Lab	Arnold *et al*., 2011 (PMID: 21982232)

Supplementary Figure 1. List of transgenic mouse strains. Transgenes used in this study are listed alphabetically, along with the type of allele, their experimental purpose, the source from which they were obtained for this study, and the papers in which the transgenes were originally described.

Antigen	Host and Isotype	Clone ID	Dilution	Immunogen	Vendor	Catalog number
APC (Adenomatous Polyposis Coli)	Mouse IgG2b	Clone CC-1	1:500	Recombinant protein consisting of amino acids 1-226 of APC	EMD Millipore, Billerica, MA	OP80
BrdU (Bromodeoxyuridine)	Rat IgG	Clone BU1/75	1:600	Chemical BrdU	Novus Biologicals, Littleton, CO	NB500-169
Cleaved caspase-3	Rabbit IgG	Polyclonal	1:1000	Synthetic peptide corresponding to amino-terminal residues adjacent to Asp175 in human caspase-3	Cell Signaling Technology, Danvers, MA	9661
Doublecortin	Rabbit IgG	Polyclonal	1:400	Synthetic peptide corresponding to human doublecortin	Cell Signaling Technology, Danvers, MA	4604
GFAP (Glial Fibrillary Acidic Protein)	Rabbit IgG	Polyclonal	1:500	GFAP isolated from cow spinal cord	Dako, Carpinteria, CA	Z0334
GFAP (Glial Fibrillary Acidic Protein)	Mouse IgG1	Clone GA5	1:1000	Purified glial filament	EMD Millipore, Billerica, MA	MAB3402
GFAP (Glial Fibrillary Acidic Protein)	Chicken IgY	Polyclonal	1:500	Full length bovine GFAP (purified from a Triton X-100 extract of myelin associated material by centrifugation and chromatography)	Abcam, Cambridge, MA	ab4674
GFP (Green Fluorescent Protein)	Rabbit IgG	Polyclonal	1:1000	Highly purified recombinant full length GFP made in Escherichia coli	Abcam, Cambridge, MA	ab6556
GFP (Green Fluorescent Protein)	Chicken IgY	Polyclonal	1:1000	Purified recombinant green fluorescent protein (GFP)	Aves Lab, Tigard, OR	GFP1020
Ki67	Mouse IgG1	Clone B56	1:400	Human Ki-67	BD Pharmingen, San Diego, CA	556003
MBP (Myelin Basic Protein)	Rat IgG	Clone 12	1:200	Intact MBP	ABD Serotec, Raleigh, NC	MCA4095
Nestin	Mouse IgG1	Clone Rat-401	1:750	Nestin purified from embryonic rat spinal cord	EMD Millipore, Billerica, MA	MAB353
Nestin	Chicken IgY	Polyclonal	1:100	Peptide corresponding to a region of the Nestin gene product shared between the rat (AAA41119, NCBI) and human (NP_001966, NCBI) gene products.	Lifespan Biosciences, Seattle, WA	LS-C73310
NeuN (Neuronal Nuclei)	Mouse IgG1	Clone A60	1:200	Purified cell nuclei from mouse brain	EMD Millipore, Billerica, MA	MAB377
NG2	Rabbit IgG	Polyclonal	1:200	Immunoaffinity purified NG2 Chondroitin Sulfate Proteoglycan from rat	EMD Millipore, Billerica, MA	AB5320
Olig2	Rabbit IgG	Polyclonal	1:10000	Fusion protein containing N-terminus of mouse Olig2	Gift of C. Stiles, Dana-Farber Cancer Institute	N/A
Olig2	Mouse IgG2a	Clone 211F1.1	1:750	Fusion protein containing N-terminus of mouse Olig2	Gift of C. Stiles, Dana-Farber Cancer Institute	N/A
PDGFRalpha	Rabbit IgG	Clone D1E1E	1:500	Synthetic peptide corresponding to residues near the carboxy-terminal sequence of human PDGFRalpha	Cell Signaling Technology, Danvers, MA	3174
PDGFRalpha	Rabbit IgG	Polyclonal	1:1000	Affinity purified recombinant ectodomain of human PDGFRalpha	Gift of W. Stallcup, Sanford-Burnham Medical Research Institute	N/A
phospho-ser10-Histone H3	Rabbit IgG	Polyclonal	1:1000	Linear peptide corresponding to human Histone H3 at Ser10.	EMD Millipore, Billerica, MA	06-570
phospho-ser55-Vimentin	Mouse IgG2b	Clone 4A4	1:500	Synthetic MPV55 phophopeptide corresponding to Mouse phophorylated vimentin Ser55 (SLYSS-phosphoS55-PGGAYC-KLH)	MBL International, Woburn, MA	D076-3
PSA-NCAM	Mouse IgM	Clone 2-2B	1:500	Viable Meningococcus group B (strain 355)	EMD Millipore, Billerica, MA	MAB5324
RFP (Red Fluorescent Proteins, including TdTomato)	Rat IgG	Clone 5F8	1:1000	Immunogen not specified, but tested on monomeric RFPs	ChromoTek, Planegg-Martinsried, Germany	5f8
RFPs (DsRed and TdTomato)	Rabbit IgG	Polyclonal	1:1000	DsRed-Express protein	Clontech, Mountain View, CA	632496
S100	Rabbit IgG	Polyclonal	1:500	S100 isolated from cow brain	Dako, Carpinteria, CA	Z033
Sox10	Goat IgG	Polyclonal	1:100	Peptide mapping at the N-terminus of Sox-10 of human origin	Santa Cruz Biotechnology, Dallas, TX	sc-17342
Sox2	Goat IgG	Polyclonal	1:250	Peptide mapping near the C-terminus of Sox-2 of human origin	Santa Cruz Biotechnology, Dallas, TX	sc-17320
Vimentin	Mouse IgM	Clone LN-6	1:200	Human thymic nuclear extract	Sigma-Aldrich, St. Louis, MO	V2258

Supplementary Figure 2. List of primary antibodies for immunohistochemistry. Primary antibodies were used at the indicated dilutions. Where available, multiple antibodies were used to validate antibody specificity. Immunogen information is provided by the vendor.

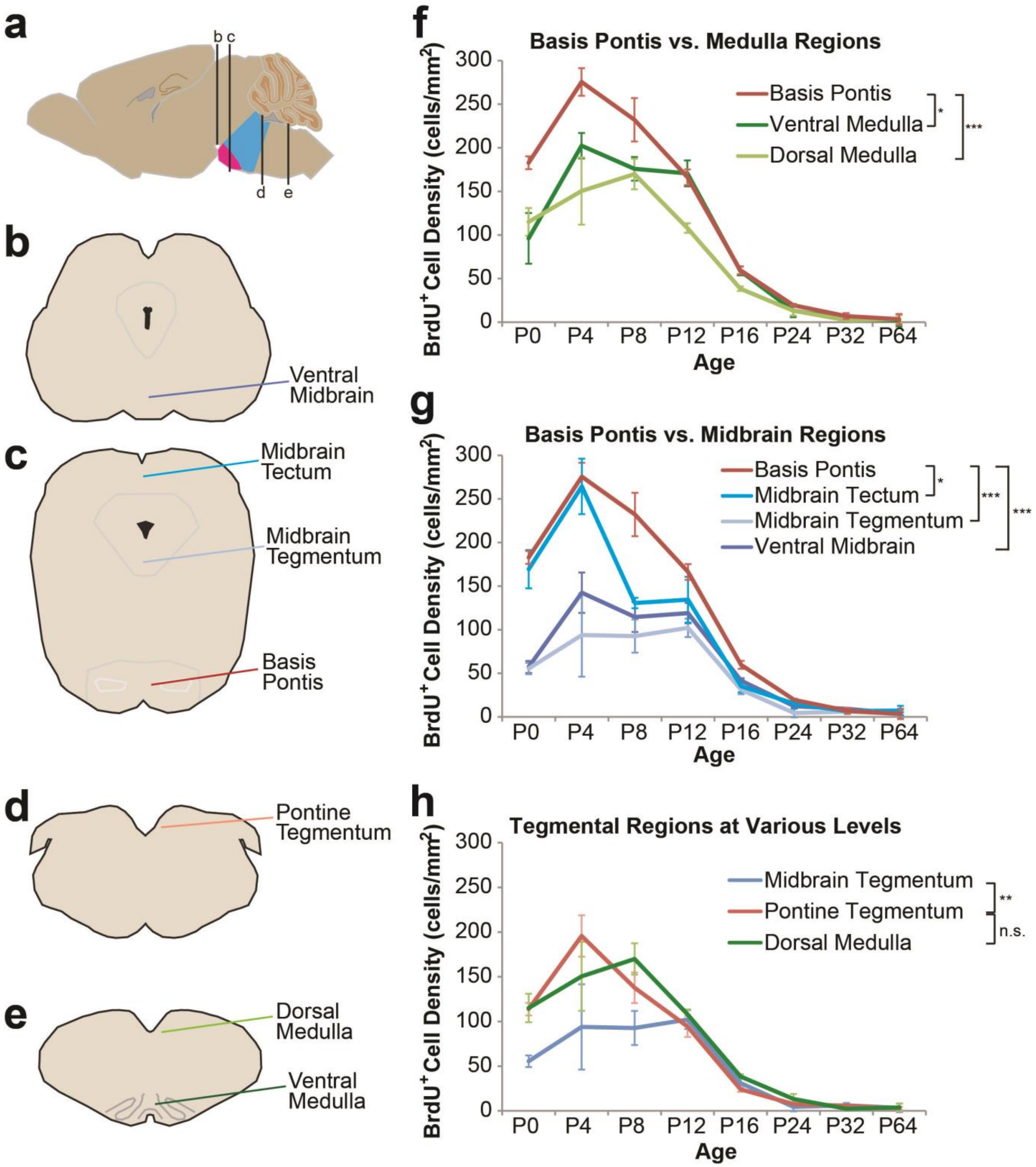

Supplementary Figure 3. Basis Pontis is the most proliferative region of postnatal brainstem.
CD1 wild type mice received a single dose of BrdU 100 min before perfusion. Immunofluorescent BrdU staining was detected by confocal microscopy. Proliferation density was computed as number of $BrdU^+$ nuclei divided by tissue area. All data points represent mean ± SEM of n = 3 mice; *P*-values based on 2-way ANOVA and Bonferroni *post hoc* adjustment. * $P < 0.05$; ** $P < 0.01$; *** $P < 0.001$; n.s. $P > 0.05$. **(a-e)** Sections in the coronal plane illustrate the levels of brainstem represented in (f-h). **(f)** Basis pontis is more proliferative than both ventral medulla ($F_{1,32}$=11.53, P = 0.0108) and dorsal medulla ($F_{1,32}$=25.14, $P < 0.0006$). **(g)** Basis pontis is more proliferative than ventral midbrain ($F_{1,32}$=78.16, $P <$ 0.0006), midbrain tegmentum ($F_{1,32}$=122.34, $P < 0.0006$), and midbrain tectum ($F_{1,32}$=8.87, P = 0.0330). **(h)** Pontine tegmentum is more proliferative than midbrain tegmentum ($F_{1,32}$=11.86, P = 0.0032), but not dorsal medulla ($F_{1,32}$=0.07, P = 0.7918).

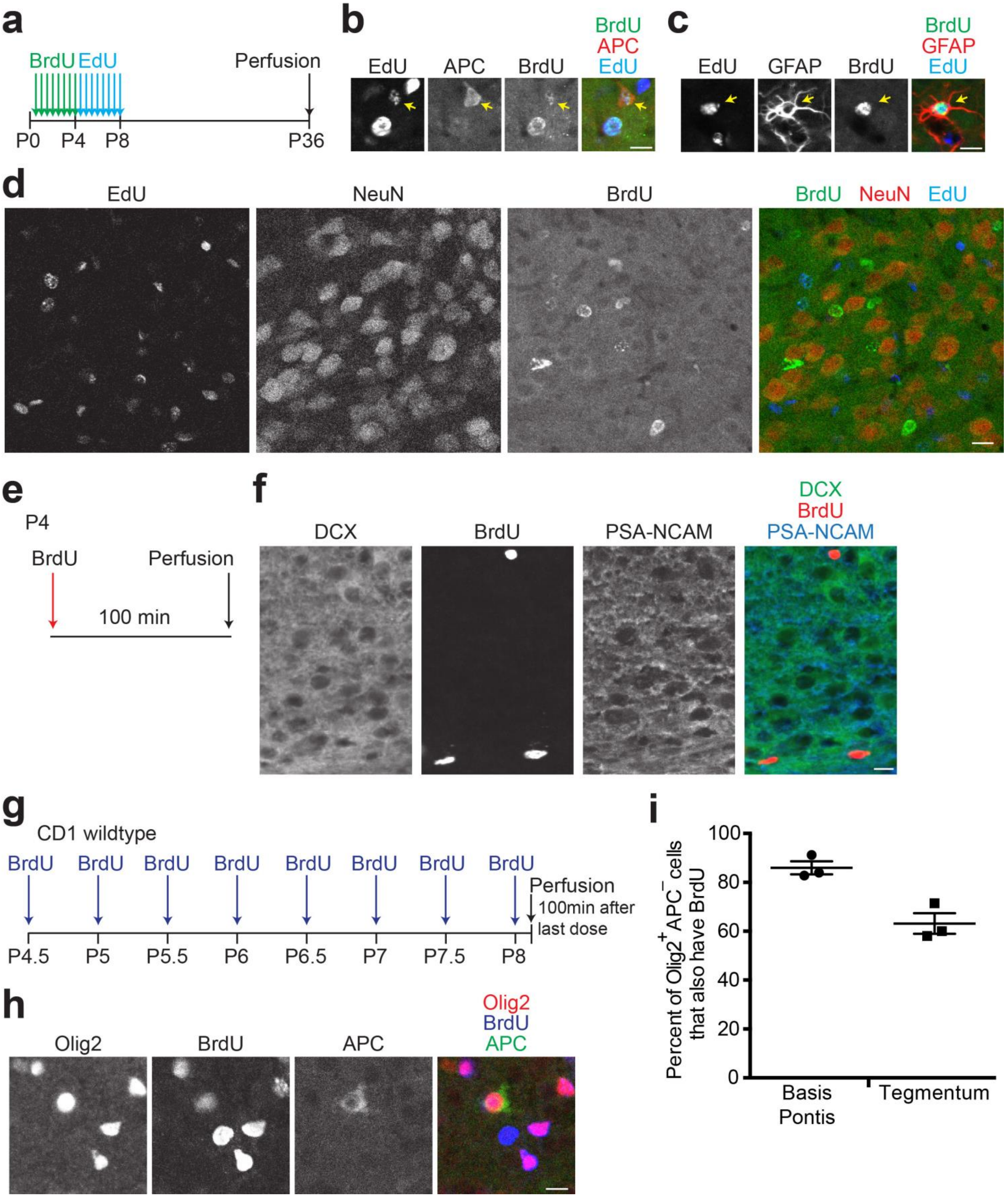

Supplementary Figure 4. Postnatal pontine proliferation is non-neurogenic. (a) CD1 wild type mice were given BrdU at 50mg/kg every 12 hrs from P0.5 through P4, then EdU at an equimolar dose (41 mg/kg) every 12 hrs from P4.5 through P8, and perfused 28 days following treatment. **(b-c)** Pons tissue from mice prepared as in (a) was stained for the indicated markers; BrdU^{+}EdU^{+} cells in basis pontis included APC^{+} oligodendrocytes and BrdU^{+}EdU^{+}GFAP^{+} astrocytes.

Scale bars = 10 µm. **(d)** Pons tissue from mice prepared as in (a) was co-stained for BrdU, EdU, and NeuN, but no $NeuN^{+}BrdU^{+}$ or $NeuN^{+}EdU^{+}$ figures were found. Shown here is basis pontis. No $NeuN^{+}XdU^{+}$ cells were observed in tegmentum either. Additional experiments involving BrdU/EdU treatment between P8.5-P16, P16.5-P24, P24.5-P32, P0 (single dose BrdU), or P4 (single dose BrdU), followed by 28-day chase period in each case, also yielded no $NeuN^{+}XdU^{+}$ pons cells. Scale bar = 10 µm. **(e)** CD1 wild-type mice were given BrdU in a single dose of 50 mg/kg 100 minutes prior to perfusion. **(f)** Pons tissue from mice prepared as in (e) was co-stained for BrdU, doublecortin (DCX), and polysialated neural cell adhesion molecule (PSA-NCAM). Shown here is basis pontis. No clear colocalization was observed between BrdU and either DCX or PSA-NCAM. Scale bar = 10 µm. **(g)** CD1 wildtype mice received a four-day course of BrdU by intraperitoneal injections at 50mg/kg every 12 hours from P4.5 to P8, and were perfused 100 minutes following the last dose of BrdU. **(h)** Pons tissue from mice prepared as in (g) was stained for BrdU, Olig2, and APC; shown here is basis pontis. Scale bar = 10 µm. **(i)** The proportion of OLPs ($Olig2^{+}$ APC^{-} cells) that were $BrdU^{+}$ after the 4-day pulse was greater in basis pontis than in tegmentum ($P = 0.0146$, unpaired *t*-test), consistent with findings of denser proliferation among basis pontis OLPs (Fig. 7d) and greater fold expansion among basis pontis oligodendroglia (Fig. 4f).

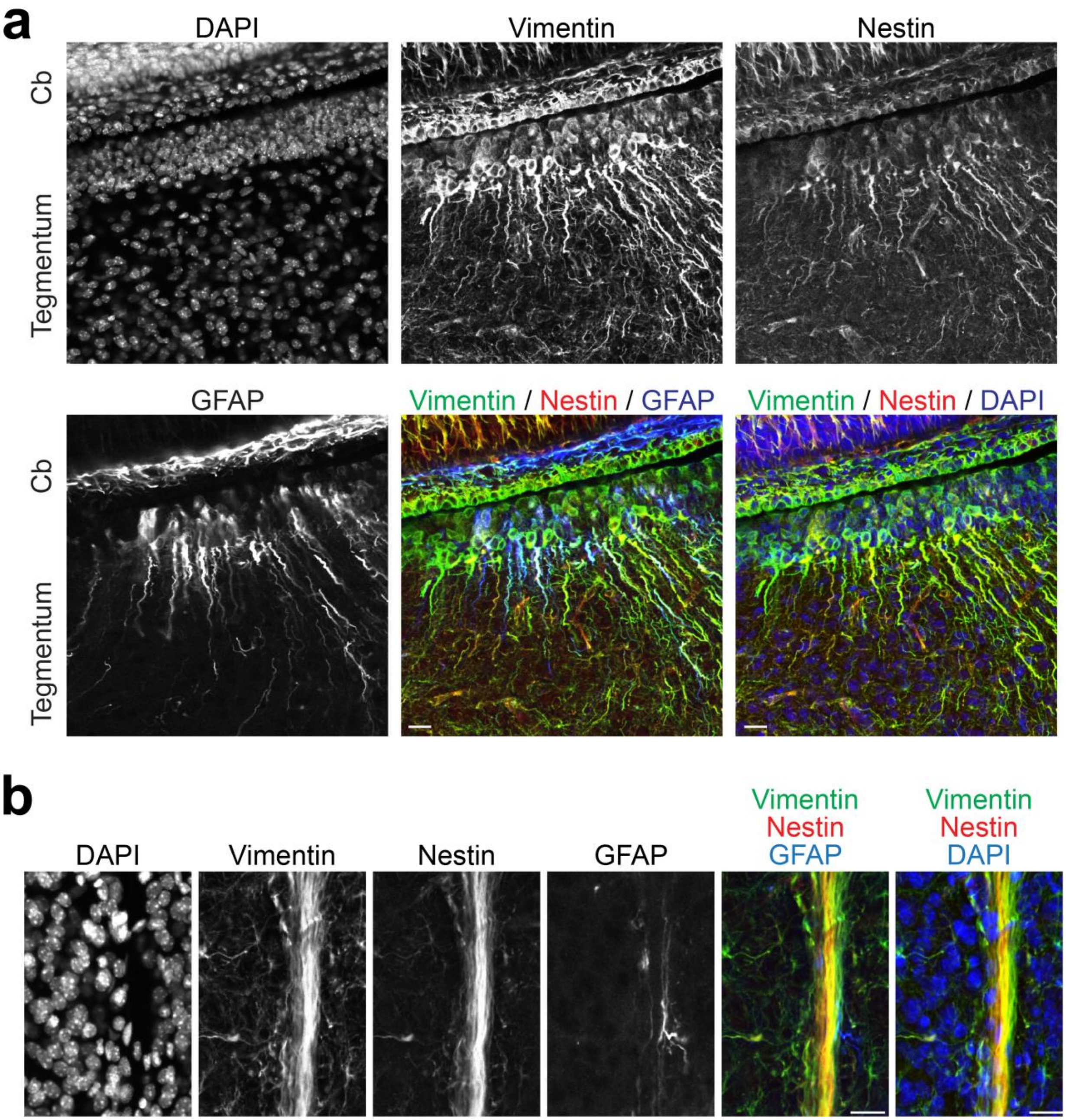

Supplementary Figure 5. Expression of intermediate filaments along 4th ventricle and midline at P4.
Sections of CD1 wildtype mouse pons tissue aged P4 were co-stained for vimentin, nestin, and GFAP, revealing partly overlapping expression patterns among the three intermediate filaments. **(a)** Radial glial fibers visible along the 4th ventricle, extending into tegmentum. Cb = Cerebellum. Scale bar = 20 µm. **(b)** Vimentin and nestin are expressed intensely in the fibers of the midline, with adjacent GFAP^{+} processes, and some filamentous Vimentin and Nestin staining within parenchyma. Scale bar = 20 µm.

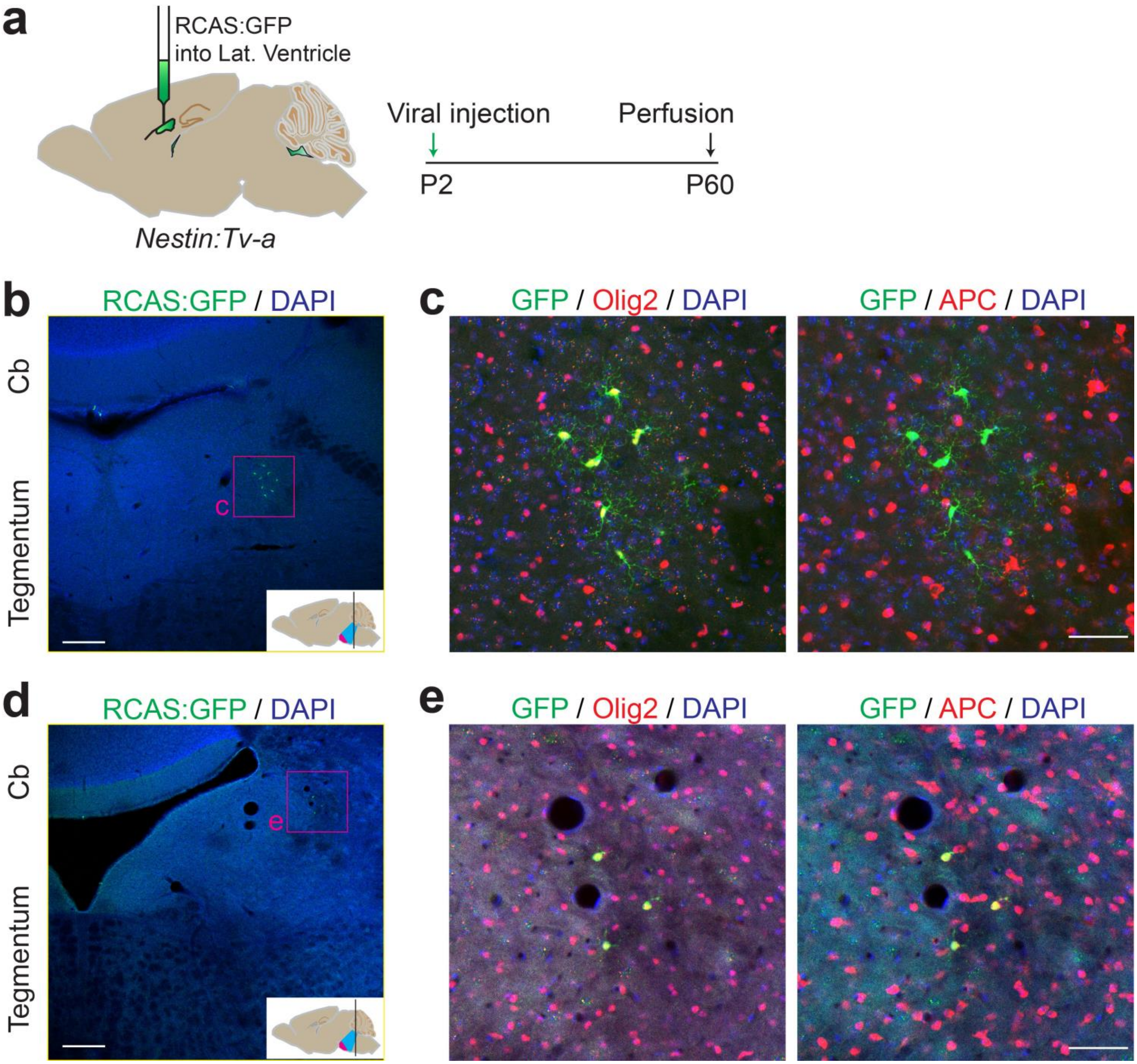

Supplementary Figure 6. Retroviral lineage tracing from Nestin^{+} VZ progenitors.
(a-e) RCAS:GFP retrovirus injected into P2 lateral ventricle of *Nestin-tva* mice resulted in sparse clusters of GFP^{+} cells in P60 pons parenchyma. **(b-c)** A cluster of GFP^{+}Olig2^{+}APC^{-} cells, with OLP-like morphology, near locus ceruleus. **(d-e)** A cluster of round, Olig2^{+}APC^{+} cells in caudal pontine tegmentum, indicating mature pons OLs deriving from postnatal 4th ventricle progenitors. Cb = Cerebellum. Scale bars = 200 µm (b,d), 50 µm (c,e).

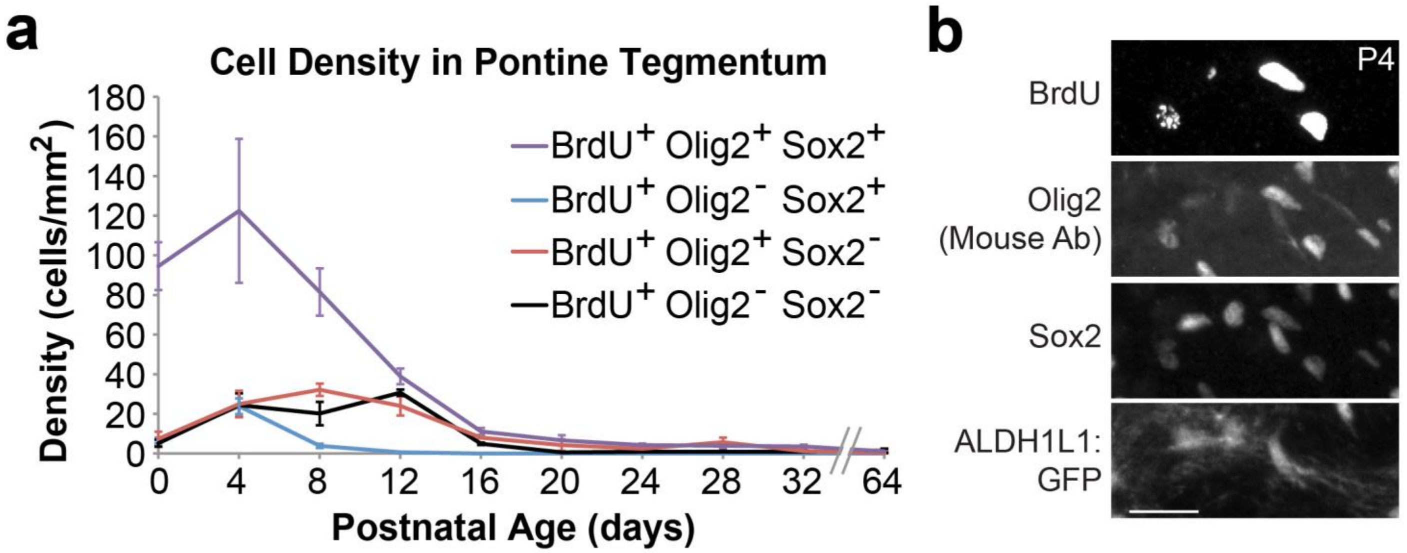

c

Cell density (cells / mm^2)

	Basis Pontis		Pontine Tegmentum	
**Age**	**BrdU$^+$GFP$^+$**	**Olig2$^+$BrdU$^+$GFP$^+$**	**BrdU$^+$GFP$^+$**	**Olig2$^+$BrdU$^+$GFP$^+$**
P0	69.4 ± 7.9	6.1 ± 3.7	22.6 ± 6.1	13.7 ± 3.8
P2	74.9 ± 8.0	3.8 ± 2.4	27.1 ± 7.2	8.3 ± 3.2
P4	73.0 ± 3.9	1.3 ± 0.9	12.4 ± 2.9	3.2 ± 1.2
P6	37.7 ± 1.2	2.8 ± 1.1	6.6 ± 0.1	0.6 ± 0.6
P8	19.6 ± 2.3	1.9 ± 1.0	1.0 ± 0.1	0.4 ± 0.2
P10	14.8 ± 3.2	0 ± 0	0.4 ± 0.2	0.1 ± 0.1
P12	2.9 ± 1.4	0 ± 0	0.3 ± 0.3	0 ± 0
P16	0 ± 0	0 ± 0	0 ± 0	0 ± 0
P20	0 ± 0	0 ± 0	0 ± 0	0 ± 0
P64	0 ± 0	0 ± 0	0 ± 0	0 ± 0

Supplementary Figure 7. Pontine Sox2$^+$ Olig2$^+$ BrdU$^+$ progenitors are predominantly not astrocytes.
(a) Classification of BrdU$^+$ cells in pontine tegmentum (basis pontis shown in Fig. 8d) by Sox2 and Olig2 status. In pairwise comparisons between the Sox2$^+$Olig2$^+$BrdU$^+$ population and each other population, 3-factor ANOVAs based on cell type, region, and age revealed that the Sox2$^+$Olig2$^+$BrdU$^+$ population is the most abundant ($F_{1,80} \geq 170.26$, $P < 0.0003$). Comparing cell types pairwise within tegmentum, 2-factor ANOVAs based on cell type and age similarly showed the Sox2$^+$Olig2$^+$BrdU$^+$ population to be greater than any other population in tegmentum ($F_{1,40} \geq 38.07$, $P < 0.0003$). P values include Bonferroni post-hoc adjustment. **(b)** Representative field of P4 *ALDH1L1:GFP* mouse pons stained for GFP, BrdU, Sox2, and a different Olig2 antibody from that used in Figure 8g, confirming that Olig2 is commonly expressed in BrdU$^+$Sox2$^+$ALDH1L1:GFP$^-$ cells. Scale bar = 20 µm. **(c)** Proliferation density of Olig2$^+$ALDH1L1:GFP+BrdU+ cells through postnatal development, listed here numerically because counts were barely visible in graph of Figure 8i, indicating that astrocytes do not account for the Sox2$^+$Olig2$^+$BrdU$^+$ population observed in wild type mice (see (a) above and Fig. 8i). Furthermore, the Olig2$^+$ subpopulation is a small proportion of total ALDH1L1:GFP$^+$BrdU$^+$ cells, except in P0 tegmentum.

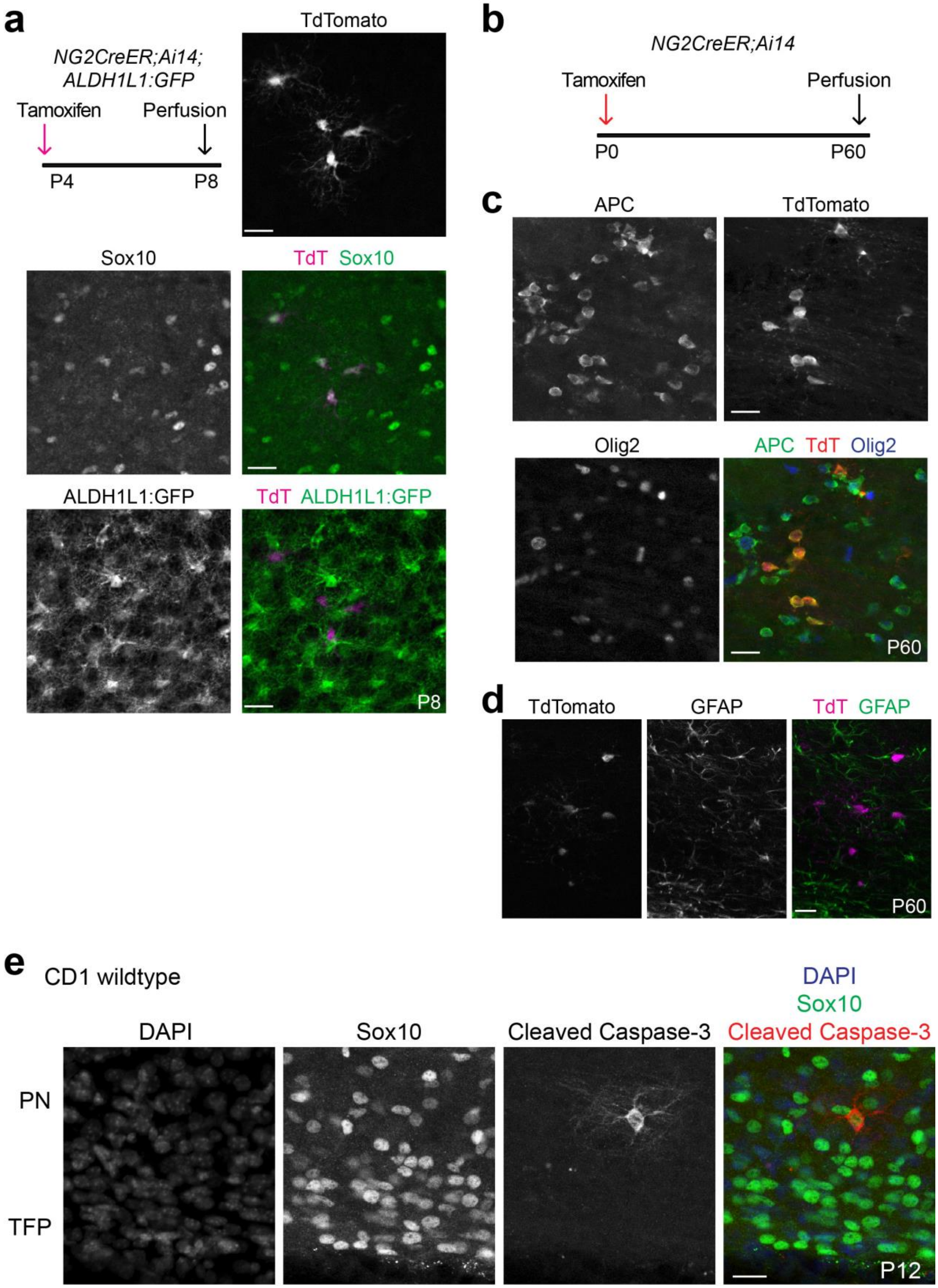

Supplementary Figure 8. Lineage restriction and cell death of pontine oligodendroglia.
(a) *NG2CreER;Ai14;ALDH1L1:GFP* mice received tamoxifen at P4 and perfusion at P8, revealing TdTomato expression in Sox10$^+$ oligodendroglia but not in ALDH1L1:GFP$^+$ astrocytes. Similar results were obtained with tamoxifen at P0. **(b-d)** Long-term fate-mapping of *NG2CreER;Ai14* mice: Animals received tamoxifen at P0 and were perfused at P60. **(c)** A cluster of TdT$^+$Olig2$^+$APC$^+$ OLs labeled in P60 *NG2CreER;Ai14* mice after tamoxifen at P0. **(d)** TdT does not colocalize

with fibers of $GFAP^+$ astrocytes in P60 *NG2CreER;Ai14* mice after tamoxifen at P0. **(e)** Apoptotic oligodendrocyte in postnatal mouse pons. CD1 wildtype mouse pons tissue aged P12 was co-stained for cleaved caspase-3 and Sox10. Note the complex, multipolar morphology of cleaved caspase-3 surrounding a $Sox10^+$ nucleus. TFP = transversus fasciculus pontis, PN = pontine nuclei. All images shown are from basis pontis. All scale bars are 20 μm.

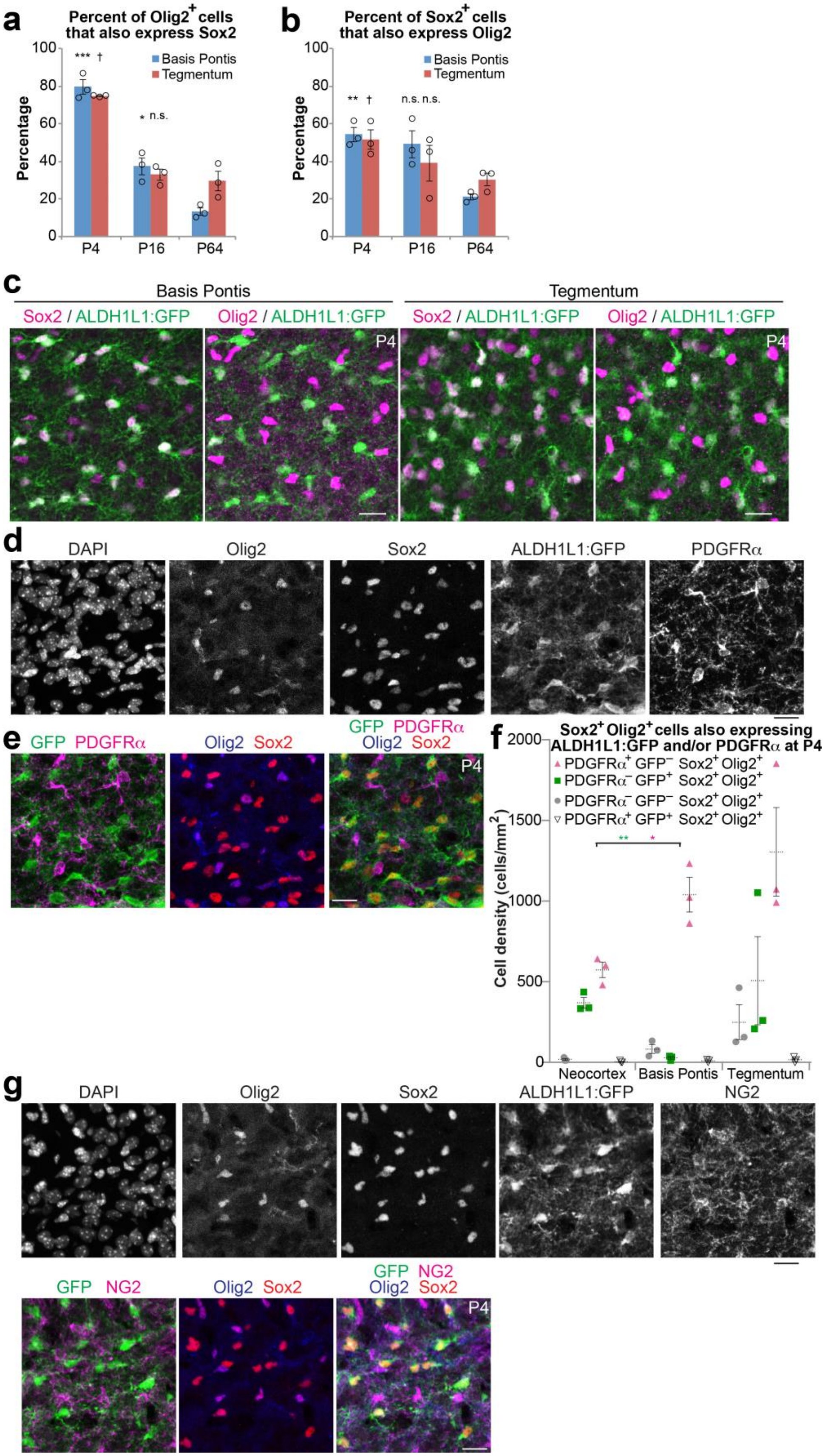

Supplementary Figure 9. Pontine Sox2$^+$ Olig2$^+$ progenitors predominantly express markers of OLPs.

(a-b) Sox2$^+$Olig2$^+$ double-positive cells constitute a notable percentage of Olig2 cells **(a)** and Sox2 cells **(b)** in wildtype mouse pons, particularly in early postnatal life. n = 3 mice per cohort; mean ± SEM; unpaired *t*-test vs. P64 basis pontis: * *P* < 0.05, ** *P* < 0.01, *** *P* < 0.001; student's t-test vs. P64 tegmentum: † *P* < 0.05. **(c)** Representative fields from P4 *ALDH1L1:GFP* pons regions co-stained for Sox2, Olig2, and the astrocyte-specific ALDH1L1:GFP reporter, revealing expression of Sox2 in many GFP$^+$ cells, and expression of Olig2 in a subset of GFP$^+$ cells (tegmentum > basis pontis). **(d)**

Representative field from P4 *ALDH1L1:GFP* basis pontis co-stained for Sox2, Olig2, GFP (marking astrocytes), and PDGFR□ (marking OLPs). **(e)** Merges of single channels from (d) show that Olig2$^+$Sox2$^+$ cells in basis pontis generally colocalize with PDGFR□ rather than ALDH1L1:GFP. **(f)** Classification of Sox2$^+$Olig2$^+$ cells in P4 *ALDH1L1:GFP* brain regions by GFP and PDGFR□ status. Shown is the density of each type of Sox2$^+$Olig2$^+$ cell. Sox2$^+$Olig2$^+$PDGFR□$^+$ OLPs are denser in basis pontis than in neocortex (unpaired t-test, $P = 0.0331$), and account for ~90% of basis pontis Sox2$^+$Olig2$^+$ cells. Graph shows individual replicates with mean ± SEM of n = 3 mice; unpaired t-test, * $P < 0.05$, ** $P < 0.01$; colors of asterisks indicate cell population being compared. **(g)** Representative field from P4 *ALDH1L1:GFP* basis pontis co-stained for Sox2, Olig2, GFP (marking astrocytes), and NG2 protein (marking OLPs); Olig2$^+$Sox2$^+$ cells in basis pontis generally colocalize with NG2 rather than ALDH1L1:GFP. All scale bars = 20 µm.

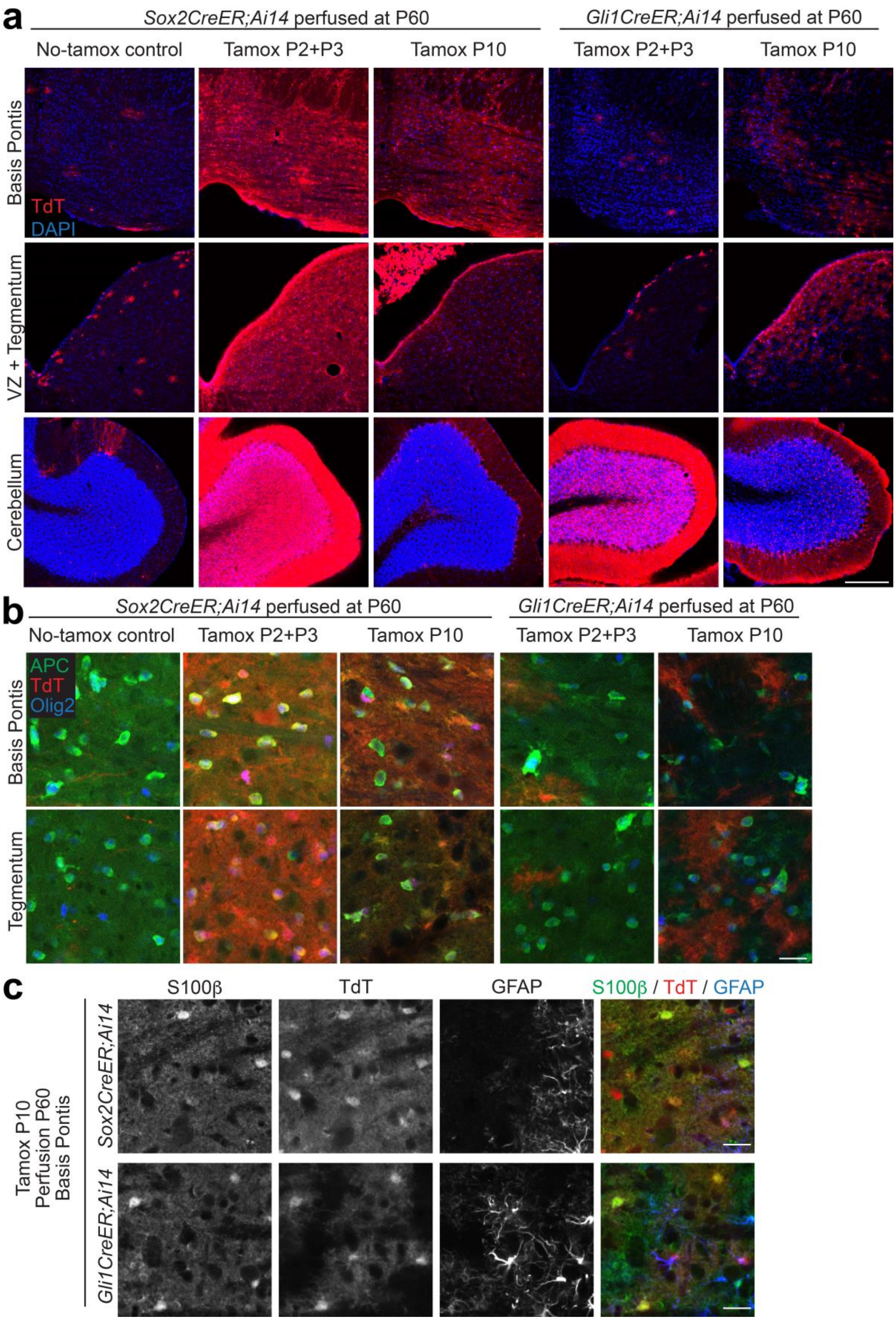

Supplementary Figure 10. Comparison of adult pons labeling by postnatal *Sox2CreER* and *Gli1CreER* recombination.

Sox2CreER;Ai14 mice or *Gli1CreER;Ai14* mice were given tamoxifen at the indicated ages and perfused at P60. Hindbrain sections were prepared and stained for TdTomato to reveal extent of adult tissue derived from postnatal Sox2$^+$ or Gli1$^+$ progenitors. **(a)** Both reporters showed extensive TdT expression in the cerebellar granule layer, as expected, with broader labeling at P2+P3 than at P10. *Sox2CreER* produced broad labeling throughout pons parenchyma, with

more at P2+P3 than at P10, while *Gli1CreER* produced sparse parenchymal labeling, and more at P10 than at P2+P3. A low rate of spontaneous recombination, producing sparse clones of cells, was observed in no-tamoxifen control *Sox2CreER;Ai14* mice. *Gli1CreER;Ai14* no-tamoxifen control mice showed no background recombination (not shown). Scale bar = 200 µm. **(b)** Higher-magnification view of pons parenchyma, stained for TdT (red), APC (green), and Olig2 (blue). TdT$^+$APC$^+$Olig2$^+$ cells were observed in *Sox2CreER;Ai14* pons, but not in *Gli1CreER;Ai14* pons. Scale bar = 20 µm. **(c)** Mice of the indicated genotypes received tamoxifen at P10 and were perfused at P60; pons sections were stained for TdT (red) and the astrocyte markers S100□ (green) and GFAP (blue). Scale bar = 20 µm.
